# Rapid coupling between solid earth and ice volume during the Quaternary

**DOI:** 10.1038/s41598-021-84448-7

**Published:** 2021-03-11

**Authors:** Yusuke Kuwahara, Kazutaka Yasukawa, Koichiro Fujinaga, Tatsuo Nozaki, Junichiro Ohta, Honami Sato, Jun-Ichi Kimura, Kentaro Nakamura, Yusuke Yokoyama, Yasuhiro Kato

**Affiliations:** 1 Department of Systems Innovation, School of Engineering, The University of Tokyo, 7-3-1 Hongo, Bunkyo-ku, Tokyo 113-8656 Japan; 2 Frontier Research Center for Energy and Resources, School of Engineering, The University of Tokyo, 7-3-1 Hongo, Bunkyo-ku, Tokyo 113-8656 Japan; 3 Ocean Resources Research Center for Next Generation, Chiba Institute of Technology, 2-17-1 Tsudanuma, Narashino, Chiba 275-0016 Japan; 4 Submarine Resources Research Center, Research Institute for Marine Resources Utilization, Japan Agency for Marine-Earth Science and Technology, 2-15 Natsushima-cho, Yokosuka, Kanagawa 237-0061 Japan; 5 Department of Planetology, Graduate School of Science, Kobe University, 1-1 Rokkodai-cho, Nada-ku, Kobe, Hyogo 657-8501 Japan; 6 Volcanos and Earth’s Interior Research Center, Research Institute for Marine Geodynamics, Japan Agency for Marine-Earth Science and Technology, 2-15 Natsushima-cho, Yokosuka, Kanagawa 237-0061 Japan; 7 Atmosphere and Ocean Research Institute, The University of Tokyo, 5-1-5 Kashiwanoha, Kashiwa, Chiba 277-8564 Japan; 8 Department of Earth and Planetary Science, Graduate School of Science, The University of Tokyo, 7-3-1 Hongo, Bunkyo-ku, Tokyo 113-8656 Japan; 9 Graduate Program on Environmental Sciences, Graduate School of Arts and Sciences, The University of Tokyo, 3-8-1 Komaba, Meguro-ku, Tokyo 153-8902 Japan; 10 Biogeochemistry Research Center, Research Institute for Marine Resources Utilization, Japan Agency for Marine-Earth Science and Technology, 2-15 Natsushima-cho, Yokosuka, Kanagawa 237-0061 Japan; 11 Department of Geosciences, University of Padova, Via G. Gradenigo 6, Padova, 35131 Italy

**Keywords:** Palaeoceanography, Palaeoclimate, Marine chemistry, Geochemistry, Sedimentology

## Abstract

The solid earth plays a major role in controlling Earth’s surface climate. Volcanic degassing of carbon dioxide (CO_2_) and silicate chemical weathering are known to regulate the evolution of climate on a geologic timescale (> 10^6^ yr), but the relationship between the solid earth and the shorter (< 10^5^ yr) fluctuations of Quaternary glacial–interglacial cycles is still under debate. Here we show that the seawater osmium isotope composition (^187^Os/^188^Os), a proxy for the solid earth’s response to climate change, has varied during the past 300,000 years in association with glacial–interglacial cycles. Our marine Os isotope mass-balance simulation reveals that the observed ^187^Os/^188^Os fluctuation cannot be explained solely by global chemical weathering rate changes corresponding to glacial–interglacial climate changes, but the fluctuation can be reproduced by taking account of short-term inputs of (1) radiogenic Os derived from intense weathering of glacial till during deglacial periods and (2) unradiogenic Os derived from enhanced seafloor hydrothermalism triggered by sea-level falls associated with increases of ice sheet volume. Our results constitute the first evidence that ice sheet recession and expansion during the Quaternary systematically and repetitively caused short-term (< 10^5^ yr) solid earth responses via chemical weathering of glacial till and seafloor magmatism. This finding implies that climatic changes on < 10^5^ yr timescales can provoke rapid feedbacks from the solid earth, a causal relationship that is the reverse of the longer-term (> 10^6^ yr) causality that has been conventionally considered.

## Introduction

The solid earth has affected and responded to climate changes throughout Earth’s history. On a geological timescale, carbon cycling in Earth’s surficial (ocean, atmosphere, biosphere, and soils) and solid earth (continental and mantle rocks) systems is driven primarily by volcanic degassing of CO_2_ and chemical weathering of silicates. These processes regulate long-term (> 10^6^ yr) climatic trends and maintain the carbon balance in Earth’s surficial system^1,2^, keeping Earth a habitable planet. In contrast, on shorter (< 10^5^ yr) timescales, the causality between the solid earth and climate changes evinced by the Earth’s surficial systems has yet to be characterised.

The environmental changes during Quaternary glacial–interglacial cycles include changes in temperature, precipitation patterns, and the atmospheric partial pressure of CO_2_ (*p*CO_2_)^3,4^ that reflect the growth and regression of ice sheets in the northern high latitudes on timescales of 10^4^–10^5^ yr. Until recently, only the carbon cycling in Earth’s surficial system was believed to respond to these cyclic environmental changes, but recent studies argue that the solid earth also responds to glacial–interglacial cycles and may affect atmospheric *p*CO_2_ as well^5–7^.

Radiogenic isotopes are useful tools for investigating solid earth responses to climate changes. Despite efforts to reconstruct Quaternary records of various isotopes (e.g. Sr, Pb, Nd, and Be) in seawater^8–10^, however, at global scale, few clear signatures of the glacial–interglacial variations of these proxies have been identified^9,10^, at least partly because the residence time of these isotopes in seawater is either too long or too short to capture global-scale variations on a timescale of 10^4^–10^5^ yr. Although seawater records of Pb and Li isotopes show distinct fluctuations associated with glacial–interglacial cycles^8,11^, the signatures are local, not global.

Here we focus on the Os isotope ratio ^187^Os/^188^Os in seawater. This value reflects the relative intensity of two dominant influxes to the ocean: riverine inputs from continents containing radiogenic Os (^187^Os/^188^Os =  ~ 1.4) and mantle-like materials containing unradiogenic Os (^187^Os/^188^Os =  ~ 0.12) such as deep-sea hydrothermal fluids and cosmic dust^12^ (Supplementary Fig. S1). Because the ^187^Os/^188^Os values of these two influxes differ by an order of magnitude, seawater ^187^Os/^188^Os responds sensitively to fluctuations in the relative intensities of continental (i.e. riverine) and hydrothermal Os fluxes to the ocean. The generally accepted residence time of Os in the open ocean is 25–45 kyr^12^; therefore, the seawater ^187^Os/^188^Os can reflect variation on a timescale of 10^4^–10^5^ yr^12,13^. This residence time is sufficiently longer than the timescale of global ocean circulation (~ 1 kyr) for seawater ^187^Os/^188^Os to be globally homogeneous at any given observation time. In modern seawater, ^187^Os/^188^Os is generally uniform within analytical uncertainty; for example, mean values ± 2 SD are 1.066 ± 0.038 in the East Pacific^14^, 1.057 ± 0.038 in the south-western Indian Ocean^15^, and 1.024 ± 0.031 in the north Atlantic Ocean^16^. Hence, fluctuations of seawater ^187^Os/^188^Os in the open ocean can be regarded as a global signal.

**Figure 1:**
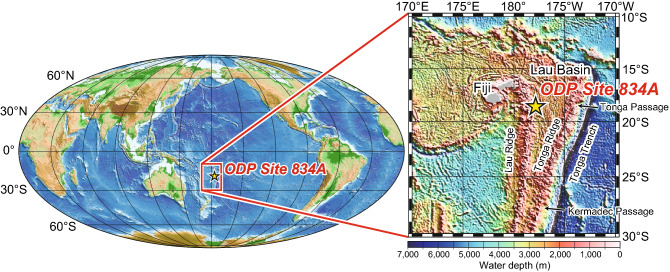
Location of the Lau Basin and Ocean Drilling Program Site 834A. Global bathymetry is based on 2-min gridded relief data (ETOPO2v2, https://www.ngdc.noaa.gov/mgg/global/etopo2.html). Detailed bathymetric data in the right panel are from ETOPO1 (https://www.ngdc.noaa.gov/mgg/global/global.html).

Even though the seawater Os isotope record is an excellent tool for reconstructing the solid earth response to glacial–interglacial cycles and various studies have analysed the Os isotope ratios of marine sediments^17–19^, the relationship between climatic cycles and the Quaternary seawater Os isotope record remains ambiguous (Supplementary Fig. S2). The ambiguity of this relationship may be due to signal distortion, caused by contamination with terrigenous detritus, or to the use of samples collected from a partly closed environment such as a semi-enclosed basin, so that a local rather than a global signal was detected^13^. Here, we analysed hydrothermal metalliferous carbonate sediments from a pelagic site to reconstruct the marine Os isotope record. Such sediments contain large amounts of Fe-oxyhydroxide, which efficiently absorbs Os from seawater and is rapidly deposited without significant contamination with continental detrital materials^20^. The Os isotope ratio in hydrothermal metalliferous carbonates captures the signature of ambient seawater, and it does not reflect the signal of the hydrothermal fluid^21^. We reconstructed the seawater Os isotope record, together with the planktonic foraminiferal radiocarbon (^14^C) and stable oxygen isotope (δ^18^O) records, used to determine depositional age, using samples collected from Ocean Drilling Program (ODP) Site 834A in the Lau Basin in the South Pacific Ocean (Fig. 1), where the sedimentary sequence covers the past 300 kyr, from marine isotope stage (MIS) 8 to the present (MIS 1) (Supplementary Fig. S3).

**Figure 2:**
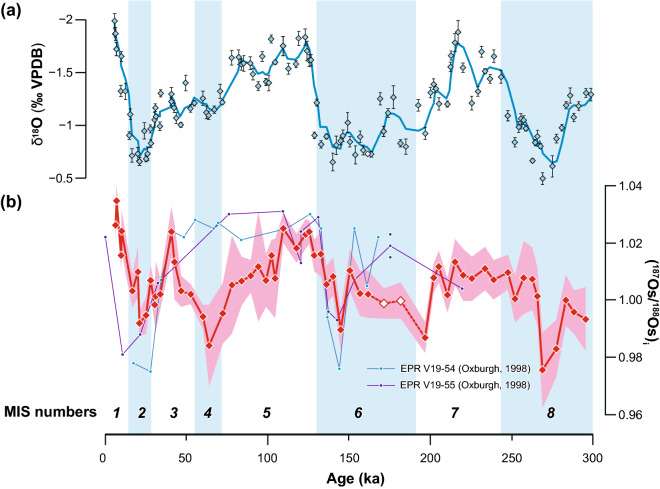
High-resolution geochemical records for the past 300 kyr from ODP Site 834A. (**a**) δ^18^O record of planktonic foraminifera. VPDB refers to the Vienna Pee Dee Belemnite standard. Diamonds indicate δ^18^O values of each sample, and the solid blue line is the 3-point moving average. Error bars indicate 2 standard deviations (SD). The open diamond at 162 ka indicates the sample for which we could not calculate a standard deviation; it is therefore used just for reference. (**b**) Seawater (^187^Os/^188^Os)_*i*_ record based on the bulk sediment analysis (red line). Red shading indicates the error range within 2 SD. The open diamonds at 171 and 182 ka indicate samples collected from the reduction halo (see “Methods” section), which were not used in the quantitative analysis. In panel (**b**), (^187^Os/^188^Os)_*i*_ records from metalliferous sediments of the East Pacific Rise (EPR) are also shown^17^. The vertical blue bars indicate glacial intervals (MIS 2, 4, 6, and 8). MIS boundaries are based on the LR04 benthic stack^22^. For the sample analysed in duplicate (18 ka), the result with the smaller uncertainty is plotted as the (^187^Os/^188^Os)_*i*_ datum. The age uncertainty (2 SD) of the samples younger than 44 ka (depositional ages determined by radiocarbon dating) is within each symbol.

Figure 2 shows high-resolution records of δ^18^O (Fig. 2a) and initial Os isotopic ratios (^187^Os/^188^Os)_*i*_ (Fig. 2b, red line) of sediments from ODP Site 834A. Note that our samples are young enough for the influence of radiogenic ^187^Os derived after sediment deposition from ^187^Re (half-life, 4.16 × 10^10^ yr) to be ignored (Supplementary Fig. S1). We determined the depositional ages of samples younger than 44 ka by radiocarbon dating (Supplementary Data S1). The radiocarbon age of the core-top sample was ~ 6 ka. We determined the depositional ages of samples older than 44 ka by correlating our δ^18^O record with a reference δ^18^O curve^22^ (see “Methods” section; Supplementary Fig. S4; Supplementary Data S2). The measured (^187^Os/^188^Os)_*i*_ values for the past 300 kyr fluctuated between 0.976 and 1.035 (average, 1.006), and that of the core-top sample (1.026) is close to that of modern seawater (^187^Os/^188^Os =  ~ 1.06)^14–16^. A strong positive correlation was observed between Os/Al_2_O_3_ and Fe_2_O_3_*/Al_2_O_3_ (Supplementary Fig. S5a), which suggests that the Os in our samples had been absorbed by Fe-oxyhydroxides in the sediments. On the other hand, the age profiles of Fe_2_O_3_*/Al_2_O_3_ and MnO/Al_2_O_3_ throughout the past 300 kyr (Supplementary Fig. S6a, b) do not show any prominent peak except for the excursion caused by the development of a “reduction halo” at ~ 170 ka^23^ (see “Methods” section). This result indicates that the supply of hydrothermal components to the study site did not fluctuate greatly. In addition, no correlation of (^187^Os/^188^Os)_*i*_ with Fe or Mn abundance was observed in the sediment (Supplementary Fig. S5b, c). These observations exclude the possibility that the (^187^Os/^188^Os)_*i*_ values can be explained by a simple mixture of ambient seawater (^187^Os/^188^Os =  ~ 1.0) and hydrothermal fluid enriched in Fe and Mn (^187^Os/^188^Os =  ~ 0.12). Therefore, Os in the metalliferous carbonate sediments from the Lau Basin is not hydrothermal Os but seawater-derived Os, and it has not been affected by local fluctuations in the abundances of hydrothermal components in the sediment.

**Figure 3:**
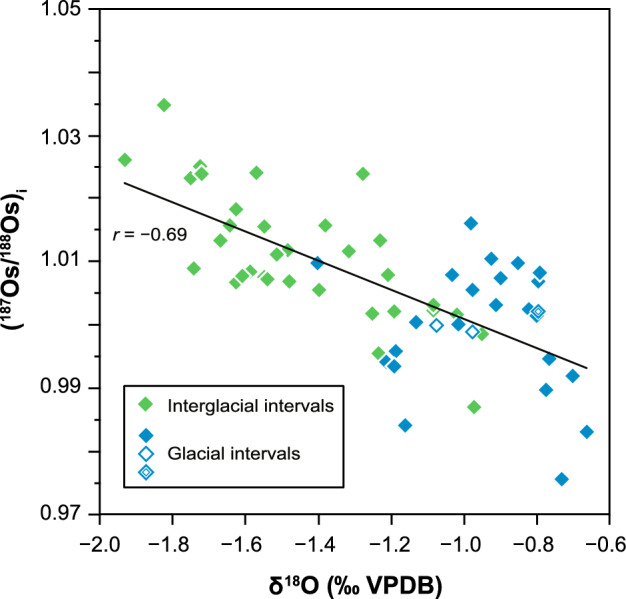
Relationship between seawater (^187^Os/^188^Os)_*i*_ based on the bulk sediment analysis and smoothed δ^18^O data of planktonic foraminifera. The plotted δ^18^O data have been smoothed by calculating the 3-point moving average for each sample. For the sample analysed in duplicate, the (^187^Os/^188^Os)_*i*_ value with the smaller uncertainty is plotted. Green and blue diamonds indicate data for interglacial (MIS 1, 3, 5, and 7) and glacial (MIS 2, 4, 6, and 8) intervals, respectively. The solid black line was fitted by linear regression to the data (*r* =  − 0.69). VPDB refers to the Vienna Pee Dee Belemnite standard. The two open blue diamonds (samples from the reduction halo; see “Methods” section) and the double square (the sample for which we could not calculate the standard deviation of δ^18^O) were excluded from the regression analysis.

During glacial periods (MISs 2, 4, 6, and 8; blue shading in Fig. 2), seawater Os isotopic ratios were systematically and significantly lower, (^187^Os/^188^Os)_*i*_ =  ~ 1.000 on average, than those during interglacial periods (MIS 1, 3, 5, and 7), (^187^Os/^188^Os)_*i*_ =  ~ 1.011 on average (*p* < 0.0001; two-sample Welch’s test). Furthermore, the (^187^Os/^188^Os)_*i*_ record correlates negatively with the smoothed δ^18^O record of planktonic foraminifera (*r* =  − 0.69; Fig. 3) determined by using the same samples (see “Methods” section). These findings suggest that the seawater Os isotopic composition has assuredly changed in response to the climate changes associated with glacial–interglacial cycles.

The (^187^Os/^188^Os)_*i*_ record of the Lau Basin fluctuates within the similar range (0.97–1.03) and with a similar fluctuation pattern during MIS 1–3 as the Quaternary seawater Os record previously reconstructed from metalliferous sediments of the East Pacific Rise (EPR)^17^ (Fig. 2b). In addition, both records show a negative excursion of ^187^Os/^188^Os at ~ 145 ka. The similarity between the Lau Basin and the EPR records suggests that the decreases of ^187^Os/^188^Os in MIS 2 and MIS 6 (especially at ~ 145 ka) may be widespread signals, at least in the South Pacific. However, in the Lau Basin sediments (^187^Os/^188^Os)_*i*_ shows an obvious decrease in MIS 4, whereas it does not in the EPR sediments. Also, in the EPR records, values from before ~ 145 ka, especially in MIS 5, are higher (~ 1.02) than those in the Lau Basin record. Possible reasons for these discrepancies include, for example, differences in the time resolution of the samples. In particular, possible uncertainties in the age models might be responsible for the presence or absence of the ^187^Os/^188^Os excursion value in MIS 4. On the other hand, the ^187^Os/^188^Os differences in MIS 5 (typically 0.02 to 0.03) between the Lau and EPR records are comparable to the spatial variability of ^187^Os/^188^Os in the modern ocean (~ 0.05;^12^).

To constrain various scenarios for explaining the measured seawater (^187^Os/^188^Os)_*i*_ record, we employed a one-box ocean Os isotope mass-balance model (see “Methods” section). Although it has been argued that the isotopic composition (including ^187^Os/^188^Os) of the radiogenic continental flux is not constant^12^, our calculations show that riverine ^187^Os/^188^Os fluctuations due to differences in the exposed lithology between glacial and interglacial intervals range from 1.41 to 1.43. This variability of less than 1.6% with respect to the modern value is too small to explain the observed seawater (^187^Os/^188^Os)_*i*_ fluctuations (see “Methods” section, Supplementary Fig. S7). Moreover, even when we used 25 kyr or 45 kyr as the residence time of Os in the ocean, instead of the default value of 35 kyr, in the simulation, the calculated results did not reproduce the observed result. Therefore, the main explanation for the observed (^187^Os/^188^Os)_*i*_ fluctuations must be changes in the relative intensities of the two dominant Os fluxes.

It has also been argued that fluctuations of the seawater Os isotope ratio during the Quaternary reflect changes in the silicate weathering rate^17,18^, and that silicate chemical weathering on land would thus be enhanced by warm, humid interglacial environments and reduced by cold, dry glacial environments. In that case, the seawater (^187^Os/^188^Os)_*i*_ record would be controlled by the riverine Os flux (i.e. the chemical weathering flux), which varies in accordance with the atmospheric *p*CO_2_ variation. However, in the model result, the amplitude of the riverine Os variation is significantly smaller than, and its phase is inconsistent with, the observed (^187^Os/^188^Os)_*i*_ variation (see “Methods” section, Supplementary Fig. S8, solid red lines). Even when the widely accepted residence time of Os in the ocean (25–45 kyr) is used (Supplementary Fig. S8a), the variation of *p*CO_2_ between glacial and interglacial periods (~ 100 ppm) cannot by itself generate the observed seawater (^187^Os/^188^Os)_*i*_ fluctuations.

Therefore, on the background fluctuation of the seawater Os isotopic record caused by the *p*CO_2_ variation, we superimposed two mechanisms, each of which rapidly modifies the seawater Os isotopic composition, making it (1) more radiogenic during interglacials and (2) less radiogenic during glacials. The first mechanism involves the chemical weathering of fresh, finely ground post-glacial sediments (glacial till) exposed during deglacial periods, which can lead to an intensified input of terrigenous materials into the ocean on a geologically short (~ 10^3^ yr) timescale^8,24,25^. High-latitude Precambrian shield rocks (e.g. in Canada and Scandinavia), which have a strongly radiogenic Os isotopic composition (^187^Os/^188^Os > 1.4), likely were eroded by ice sheets or glaciers during glacial periods, and as a result a large amount of glacial till would have been produced beneath the ice sheets. The glacial till exposed by deglaciation would be readily weathered because of the warmer climate and intensified hydrological cycle of the deglacial period, resulting in significant input of radiogenic Os to the ocean via rivers^8,24,25^.

Regarding the second mechanism, by which unradiogenic Os is supplied to the ocean, it has been shown that seafloor magmatism and hydrothermalism can be intensified by sea-level fluctuations caused by changes in global ice volume^7,26–28^. When the sea level falls because of ice sheet expansion, hydrostatic pressure at the seafloor is reduced, facilitating decompression melting and magma production beneath mid-ocean ridges^7,26–30^. In turn, the intensified seafloor magmatism rapidly increases the unradiogenic hydrothermal Os flux, causing the seawater ^187^Os/^188^Os to shift to less radiogenic values^26^.

**Figure 4:**
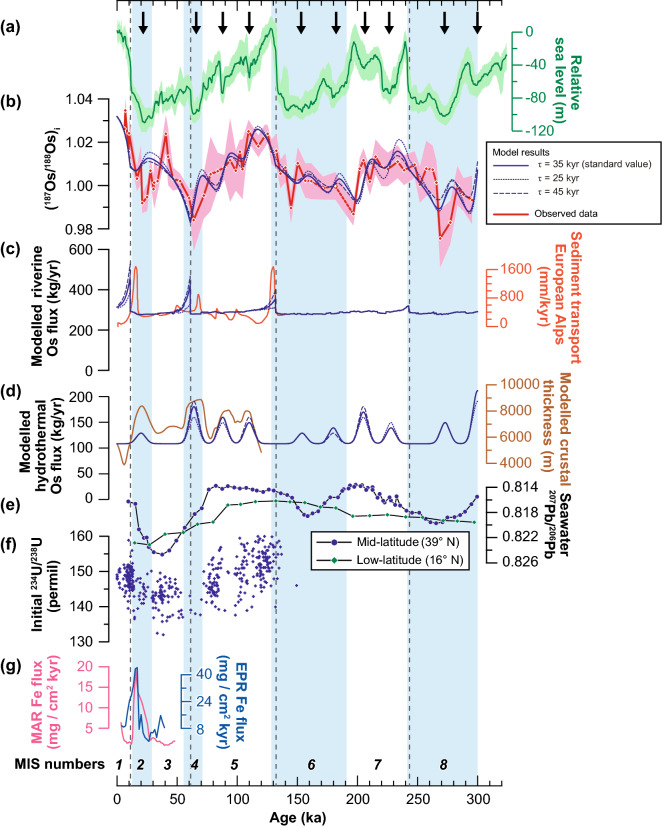
Simulated seawater (^187^Os/^188^Os)_*i*_ curve compared to other geological records. (**a**) Relative sea level of the Red Sea^31^. Green shading indicates the 95% confidence interval. (**b**) Calculated seawater (^187^Os/^188^Os)_*i*_ profile based on the best-fit scenario and the observed seawater (^187^Os/^188^Os)_*i*_ profile from ODP Site 834A. Red shading indicates 2 SD of the observed data. The two open diamonds indicate samples collected from the reduction halo. (**c**) Modelled riverine Os flux (blue line) and the sediment transport rate in the European Alps^34^ (orange line, right axis, called the equivalent denudation rate for the Alpine region in ref.^34^). (**d**) Modelled hydrothermal Os flux (blue line) and modelled seafloor crustal thickness^26^ (brown line). Seafloor crustal thickness was calculated by using the sea level curve and a dynamic model of melt production beneath mid-ocean ridges^26^. (**e**) Seawater ^207^Pb/^206^Pb record from high-latitude (39° N) (BM1969.05; blue circles) and low-latitude (16° N) (TR079 D-14; green diamonds) sites in the Atlantic^8^. (**f**) Seawater ^234^U/^238^U record^35^. (**g**) Hydrothermal Fe flux records obtained from the Mid-Atlantic Ridge^28,38^ (MAR KN207-2-GGC3; red line), and East Pacific Rise^27,38^ (EPR, Y71-09-106; blue line). In panels (**b**)–(**d**), the modelled results obtained with shorter (*τ* = 25 kyr) or longer (*τ* = 45 kyr) Os residence times compared with the standard scenario (*τ* = 35 kyr) are also shown (dotted and dashed blue lines, respectively). Vertical black dashed lines indicate the approximate timing of local maximum sea level rise rates at glacial terminations^31^, and black arrows at the top of the figure indicate the timing of local sea level minima.

We incorporated these two mechanisms generating Os flux pulses into our model and searched for the best fit given the following two constraints: (1) riverine Os pulses should occur during deglaciations (11, 61, 132, and 243 ka)^31^, and (2) hydrothermal Os pulses should coincide with minimum values of relative sea level (RSL; Fig. 4a) (> 20 m drop compared to the previous condition; 20, 64, 88, 110, 154, 180, 205, 228, 273, and 300 ka)^31^. The magnitude of the total Os input in each pulse was set to match the observed seawater (^187^Os/^188^Os)_*i*_ record (see “Methods” section).

We obtained the best fit (Fig. 4b, solid blue line) when the riverine Os pulse magnitudes were 7 × 10^5^, 6 × 10^5^, 3 × 10^5^, and 1 × 10^5^ kg (Fig. 4c, blue solid line) and the hydrothermal Os pulse magnitudes were 2 × 10^5^, 7 × 10^5^, 5 × 10^5^, 4 × 10^5^, 2 × 10^5^, 3 × 10^5^, 6 × 10^5^, 3 × 10^5^, 4 × 10^5^, and 1 × 10^6^ kg, in order from younger to older ages (Fig. 4d, solid blue line). The modelled seawater (^187^Os/^188^Os)_*i*_ record based on the default residence time of 35 kyr is generally similar to the observed record (Fig. 4b). Moreover, the results of the simulations conducted using Os residence times of 25 kyr and 45 kyr showed a pattern similar to the standard scenario (Fig. 4b, dotted and dashed blue lines, respectively; see “Methods” section). Because we prioritized simulation of the overall trend of the observed ^187^Os/^188^Os record over reproducing each short-term variation by inputs of arbitrary forcings, there are some discrepancies between observed and modelled seawater (^187^Os/^188^Os)_*i*_ in MIS 3 (~ 40 ka) and MIS 8 (~ 270 ka) (see “Methods” section).

The modern riverine Os flux from two major rivers (St. Lawrence and Mackenzie rivers) draining the area covered by the Laurentide ice sheet, which is regarded to have played a key role in Quaternary climatic cycles, is estimated to be ~ 16 kg/yr^32^. If the denudation rate of the Laurentide ice sheet area was about five times the Holocene rate during the deglaciation^33^, then the riverine Os flux at that time might have been ~ 80 kg/yr. If the deglaciation following the last glacial maximum (LGM) lasted 18 kyr, the supply capacity of glacial till from the Laurentide ice sheet area can be estimated to be ~ 1.4 × 10^6^ kg of Os. Therefore, the modelled riverine Os injection pulse for the last deglaciation, ~ 7 × 10^5^ kg, is about 50% of the estimated supply capacity of the entire Laurentide ice sheet area.

The sediment transport rate from the European Alps to surrounding sea areas (North Sea, Black Sea, and the Mediterranean) during the past 140 kyr rose significantly during deglaciations^34^ (Fig. 4c, orange line), and the timing of these rises generally coincides with the timing of our calculated riverine Os pulses. In addition, the Quaternary seawater Pb isotopic record (^207^Pb/^206^Pb) from the mid-latitude (39° N) Atlantic shows distinct fluctuations associated with glacial–interglacial cycles (Fig. 4e, blue circles), whereas that from the low-latitude Atlantic (16° N) shows much less variability^8^ (Fig. 4e, green diamonds); this result suggests that significant terrigenous input from high-latitude regions can critically affect the seawater isotopic composition. Furthermore, the seawater uranium isotope ratio (δ^234^U) was lower during the last glacial period (20–50 ka) than the modern value, and it systematically increased from the LGM to the Holocene^35,36^ (Fig. 4f). The variation in δ^234^U can also be explained, at least partly, by an increased ^234^U flux into the ocean caused by deglacial environmental changes^35–37^, similar to the mechanism causing radiogenic Os pulses. Considering these lines of geological and geochemical evidence together with the modelled riverine Os flux pulses, the rapid increases in seawater ^187^Os/^188^Os during deglacial periods can be reasonably explained by rapid inputs of riverine Os derived from the weathering of glacial till.

The weathering of glacial till may affect seawater ^187^Os/^188^Os not only through the excess supply of riverine Os but also through an increase of the ^187^Os/^188^Os value of riverine water draining the glacial area. Research on the chemical weathering of glacial moraines and the dissolution of Os into riverine water suggests that minerals with a high Re/Os ratio (Re/Os ~ 2000, with ^187^Os/^188^Os up to ~ 14) such as biotite preferentially undergo chemical weathering^24^; thus, at the initial stage of deglaciation, riverine water draining glacial areas might have higher ^187^Os/^188^Os values than the bedrock. However, even if such incongruent weathering is taken into account, our fundamental argument that deglaciation accompanied by intense chemical weathering of glacial till may cause an increase of seawater ^187^Os/^188^Os remains valid, because both mechanisms affect seawater ^187^Os/^188^Os value through the excess supply of radiogenic ^187^Os in riverine water.

The trend of the modelled hydrothermal Os flux was generally consistent with the modelled seafloor crustal thickness based on RSLs and the magma decompression dynamics during the past 120 kyr^26^ (Fig. 4d, brown line). The inferred cause of increased hydrothermal Os fluxes was intensified seafloor magmatism. The expansion of ice sheets led to falling sea levels, which in turn enhanced magma production by reducing hydrostatic pressure at the seafloor. The prominent peaks during the LGM in the mass accumulation rate of Fe in hydrothermal sediments near the Mid-Atlantic Ridge and East Pacific Rise (Fig. 4g)^27,28^ supports this hypothesis, although the relationship between the Fe content in hydrothermal sediments and the intensity of seafloor hydrothermalism is still under debate^27,28,38–40^. The hydrothermal Os pulses calculated by the model represent global increases of 20–95% compared to the baseline flux (Fig. 4e). Given that a fall in RSL during glacial periods of ~ 120 m might cause a ~ 24% increase in the magma production rate^29^ and an increase of up to 200% in the amount of hydrothermal Fe released^28^, the calculated increases in the hydrothermal Os flux are within the possible range of the glacial increase in seafloor hydrothermalism.

In this study, we have demonstrated that fluctuations of global seawater ^187^Os/^188^Os can be explained by pulsed inputs of Os, caused by intense chemical weathering of glacial till during deglacial periods^8,24,25^ and by enhanced hydrothermal activity triggered by falling sea levels^26–28^. We conclude that these two feedback mechanisms might have caused the solid earth system to respond rapidly and systematically to continental ice sheet fluctuations on a timescale of < 10^5^ yr. These feedbacks indicate that, while solid earth processes regulate climate on a longer (> 10^6^ yr) timescale as conventionally emphasized, the reverse is true for the shorter (< 10^5^ yr) term processes involving ice-volume fluctuations.

## Methods

### Study site and materials

The Lau Basin is about 3000 km east of the Australian continent and about 2000 km to the north–north-east of the North Island of New Zealand (Fig. 1). From a hydrological perspective, the Lau Basin is strongly affected by the western boundary current, which flows into the basin through the Tonga and Kermadec Passages; therefore, the water mass exchange [3–4 ± 1 Sv (1 Sverdrup = 10^6^ m^3^/s)] between the Lau Basin and the South Pacific is sufficient to produce an open ocean environment in the basin^41,42^. Because the Tonga and Kermadec Passages have a water depth of 1000–2000 m, the water mass exchange through these passages should have been sufficient even during glacial periods, when the sea level was ~ 120 m lower. Therefore, the Lau Basin has been an open ocean environment throughout the Quaternary, and sediments in the basin preserve the global seawater Os isotope signature.

The Lau Basin is an active back-arc basin where seafloor spreading, accompanied by continued hydrothermal activity, is still progressing^43–45^. Therefore, hydrothermal metalliferous sediments are widely distributed in the basin. ODP Leg 135 Site 834A is located in the western part of the basin, ~ 100 km east of the Lau Ridge. The core sediments have been divided into four units (Units I, II, III, and IV) according to their lithology^46^.

Unit I, which was deposited during the Quaternary^46,47^ (Supplementary Fig. S3a), has a hemipelagite lithology characterised by metalliferous carbonates. The unit consists mainly of nannofossil ooze containing foraminiferal shells and a large quantity (~ 10 vol.%) of opaque, brownish-coloured grains inferred to be Fe–Mn-oxyhydroxides (Supplementary Fig. S3b, c). In addition, Unit I includes interbedded turbiditic layers consisting of relatively coarse foraminiferal sand^46,48^. According to the onboard initial core description and our smear slide observations, a tephra layer occurs at 11 m below the seafloor (mbsf), and the volcanic component increases to > 10 vol.% in the core sediments adjacent to the tephra^46,48^ (Supplementary Fig. S3c, d).

Considering these observed characteristics, we collected 125 samples at 2 cm intervals from the uppermost part (0–10.8 mbsf; from 135-834A-1H-1, 1–3 cm, to 2H-3, 16–18 cm) of Unit I. The turbiditic layers were excluded from the sampling, however, because turbidite deposition is a geologically instantaneous event (Supplementary Fig. S3a). All samples were analysed for oxygen isotopes, and the 115 samples from 0 to 10.4 mbsf (from 135-834A-1H-1, 1–3 cm, to 2H-2, 132–134 cm) were analysed for bulk chemistry. Among these 115 samples, five (from 135-834A-2H-1, 99–101 cm, to 2H-1, 113–115 cm), collected from just below a turbidite layer, were orange in colour and showed significant fluctuations in the contents of redox-sensitive elements, such as Fe and Mn (Supplementary Fig. S6a). The redox-sensitive elements in this interval, which is referred to as a reduction halo^23^, might have been secondarily mobilised under reductive conditions after deposition. We therefore used the bulk chemistry and Re-Os isotope data collected from these five samples for reference only; we did not include them in our quantitative analyses because the initial geochemical characteristics of these samples may not have been preserved.

### Bulk chemical analyses

The bulk chemistry of all sediment samples, except for the 10 samples above the tephra containing large amounts of volcaniclastic materials, was analysed. The 115 samples were dried at 40 °C and pulverised in an agate mortar. The powdered samples were further dried at 110 °C for ~ 12 h. Next, loss on ignition (LOI) was determined as the weight loss during igniting at 950 °C for over 6 h. The major element concentrations were determined by X-ray fluorescence (XRF) on fused glass beads with a Rigaku 3270 spectrometer at the Ocean Research Institute, The University of Tokyo. Sample preparation and the analytical procedures are described elsewhere^49^. Trace and rare-earth element (REE) contents were measured by inductively coupled plasma-quadrupole mass spectrometry (ICP-QMS; Agilent 7500c) at the Department of Systems Innovation, The University of Tokyo, following previously described procedures^50^. The major and trace element contents and the REE patterns, normalised by post-Archean average Australian shale (PAAS^51^) values, are presented in Supplementary Data S3 and Supplementary Fig. S5g, respectively.

The high CaO contents (36.0 wt.% on average, *n* = 110) and LOI values (33.6 wt.% on average, *n* = 110) confirm that our samples were composed mainly of CaCO_3_. They also contained large amounts of Fe and Mn: The contents of Fe_2_O_3_* (total iron as Fe_2_O_3_) and MnO, calculated on a carbonate-free basis (CFB), were as high as 16.6 wt.% and 5.72 wt.%, respectively, and Al_2_O_3_-normalised contents of Fe_2_O_3_* and MnO were positively correlated (*r*^*2*^ = 0.76; Supplementary Fig. S5d, excluding reduction halo samples). These features clearly show that our samples are metalliferous carbonates (i.e. biogenic carbonate containing large amounts of Fe–Mn-oxyhydroxides). Moreover, the Al_2_O_3_-normalised contents of elements derived from seawater (e.g. ∑REY, P_2_O_5_) showed strong positive correlations (*r*^*2*^ = 0.76–0.84; excluding reduction halo samples) with Fe_2_O_3_*, which is mainly derived from hydrothermal plumes (Supplementary Fig. S5e, f). These correlations are consistent with a scavenging mechanism, by which elements are extracted from seawater by Fe-oxyhydroxides originated from seafloor hydrothermal activities^52,53^. The PAAS-normalised REE patterns show distinctly negative Ce anomalies, positive Y anomalies, and enrichment in heavy REEs (Supplementary Fig. S5g); these results also suggest that the REEs in our samples were derived mostly from the ambient seawater.

### Oxygen isotope analysis

The stable oxygen isotope composition of all samples (*n* = 125) was determined by stable isotope mass spectrometry (Thermo Finnigan MAT253) at the Center for Advanced Marine Core Research, Kochi University. Because the number of planktonic foraminifera was an order of magnitude greater than that of benthic foraminifera in our samples, for this analysis we picked planktonic foraminifer shells from the sediment to use for obtaining a high-resolution climatic record.

Twenty sand-sized (250–300 µm) planktonic foraminifer (*Globigerinoides ruber*) shells were picked from each sampling interval and cleaned ultrasonically by methanol and Milli-Q water before the analysis. The measured isotopic ratios were converted to standard delta notation (δ^18^O) relative to the Vienna Pee Dee Belemnite standard. The analytical precision (2 standard deviations, SD) was better than ± 0.15‰. Because the standard deviation of sample 135-834A-1H-4, 13–15 cm (162 ka), could not be calculated for a technical reason, oxygen isotope data from this sample were excluded from the quantitative analyses and used only for reference.

The oxygen isotopic compositions (δ^18^O) of planktonic foraminifera in our samples are presented in Supplementary Data S2.

### Age model

We obtained 26 radiocarbon dates (Supplementary Data S1) on foraminifera from samples younger than 44 ka by accelerator mass spectrometry (AMS) at the Atmosphere and Ocean Research Institute, The University of Tokyo^54^, by a previously described analytical method^55^. For samples older than 44 ka, depositional ages were determined by matching measured δ^18^O values to values on a reference curve (LR04 Benthic Stack^22^). Because the overall variation pattern of our planktonic δ^18^O curve was basically comparable to the reference curve^22^, we assumed that the inflection points of two curves corresponded, and picked 14 tie-points corresponding to obvious peaks in the LR04 stack (Supplementary Fig. S4). We then estimated the depositional ages of the samples between the tie-points by linear interpolation. As a result, the age difference between adjacent samples was generally 1–5 kyr.

### Rhenium and osmium analyses

On the basis of smear slide observations and geochemical characteristics, we selected 59 samples (including two from the reduction halo) for Re-Os isotopic analysis (Supplementary Data S4) by negative-mode thermal ionisation mass spectrometry (N-TIMS; Thermo Finnigan TRITON) at the Japan Agency for Marine-Earth Science and Technology (JAMSTEC) or by multi-collector inductively coupled plasma mass spectrometry (MC-ICP-MS; Thermo Fisher Scientific NEPTUNE) at JAMSTEC. A duplicate analysis of one sample (sample 135-834A-1H-2, 36–38 cm) by both N-TIMS and MC-ICP-MS was also performed (Supplementary Fig. S3a). All selected samples were from sediments shallower than 11 mbsf deposited during the past 300 kyr. Because the two samples collected from the reduction halo (135-834A-2H-1, 102–104 cm, 113–115 cm) may have been altered diagenetically, the data from those samples were used only for reference.

Samples were prepared for Os isotope analysis by the Carius tube digestion method^56^. About 1 g of powder from each bulk sample was used. First, carbonates were removed by the addition of a 30% HCl solution. We then added Re and Os spike solutions, for determination of Re and Os concentrations by the isotope dilution method, and 68% HNO_3_ solution. Next, 10 mL of inverse aqua regia was added to the 27 samples analysed by N-TIMS, and 4 mL of inverse aqua regia was added to the remaining samples analysed by MC-ICP-MS. Then, the samples were digested in the Carius tubes at 220 °C for 24 h. For the samples analysed by N-TIMS, after Os purification^50^, Os isotopes were measured by using the jumping mode of a secondary electron multiplier ion counter. For the samples analysed by MC-ICP-MS, the sparging method was used for Os introduction^57,58^; multiple Faraday cups were used for sample analysis; and a multiple ion counter was used for blank analysis. (^187^Os/^188^Os)_*i*_ values obtained by the duplicate analysis of sample 135-834A-1H-2, 36–38 cm, by both N-TIMS and MC-ICP-MS agreed well within error. Therefore, we inferred that the differences in sample preparation and analytical method between N-TIMS and MC-ICP-MS did not affect the analytical results^57^. The overall analytical error (2 SD) for (^187^Os/^188^Os)_*i*_ was better than 0.0191.

After the Os isotopic analysis, samples were prepared for Re isotopic analysis^57^. An ion exchange resin was used to extract Re from the inverse aqua regia solution of each sample. Then, the Re-bearing 6 M HNO_3_ solution was condensed and loaded onto a Pt filament for N-TIMS or diluted with 1 mL Milli-Q water for MC-ICP-MS. Twenty-seven samples were analysed by using the total evaporation method^59^ and N-TIMS. The rest were analysed by MC-ICP-MS with solution introduction into the glass torch by the iridium (Ir) standard addition method with mass discrimination correction^60^. All Re-Os data were corrected by subtracting procedural blank values (Os, 0.17–2.8 pg; Re, 4.6–12 pg).

We calculated the initial Os isotope ratio [(^187^Os/^188^Os)_*i*_] by subtracting radiogenic ^187^Os produced by ^187^Re internal decay from the time of deposition to the present from the measured Os isotope ratio [(^187^Os/^188^Os)_*measured*_]:

1 $$\begin{array}{*{20}c} {\left( {\frac{{^{{{{187}}}} {\text{Os}}}}{{^{{{{188}}}} {\text{Os}}}}} \right)_{{{i}}} = \left( {\frac{{^{{{{187}}}} {\text{Os}}}}{{^{{{{188}}}} {\text{Os}}}}} \right)_{{{{measured}}}} - \left( {\frac{{^{{{{187}}}} {\text{Re}}}}{{^{{{{188}}}} {\text{Os}}}}} \right) \times \left[ {{\text{exp}}\left( {{\lambda t}} \right) - {{1}}} \right]} \\ \end{array}$$

where *λ* and *t* are the decay constant of ^187^Re (*λ* = 1.666 × 10^−11^ yr^−1^)^61^ and the sample age (yr), respectively. This age correction had only a minuscule effect on (^187^Os/^188^Os)_*i*_ values of the metalliferous carbonate, because internal decay of ^187^Re after deposition had a negligible effect owing to the low ^187^Re/^188^Os values of the samples and the long half-life of ^187^Re (4.16 × 10^10^ yr).

To compare the Os contents of our samples with the bulk chemistry data, all contents were calculated on a CFB and normalised by the Al_2_O_3_ contents; data from the reduction halo samples were excluded from all correlation analyses. As a result, the Os content showed strong positive correlations with both Fe_2_O_3_* and MnO (*r*^2^ = 0.80–0.87; Supplementary Fig. S5a, h). The Os and SiO_2_ contents were not correlated, however (*r*^2^ = 0.026; Supplementary Fig. S5i), indicating that the contribution of biogenic silica to the Os content was negligible. In addition, measured seawater (^187^Os/^188^Os)_*i*_ was not correlated with the Fe_2_O_3_* or MnO contents (*r*^2^ = 0.017–0.031; Supplementary Fig. S5b, c), and (^187^Os/^188^Os)_*i*_ fluctuated systematically throughout the 300 kyr of the record, whereas the age profiles of Fe_2_O_3_*/Al_2_O_3_ and MnO/Al_2_O_3_ did not show prominent fluctuations, except for the reduction halo at ~ 170 ka (Supplementary Fig. S6a, b). These results indicate that local hydrothermal activity in the Lau Basin did not affect the ^187^Os/^188^Os values recorded in the sediments at ODP Site 834A. Because, as is well known, Os in seawater is efficiently trapped by Fe-oxyhydroxide particulates^12,20,21^, these correlation results indicate that the metalliferous carbonates in the Lau Basin scavenged Os from the seawater.

### Comparison between the ^187^Os/^188^Os and δ^18^O records

To investigate the relationship between the seawater Os isotopic composition and climate, we compared our seawater (^187^Os/^188^Os)_*i*_ data with the foraminiferal δ^18^O record obtained in this study. To focus on the overall climate trends on timescales of tens to hundreds of kiloyears, we removed high-frequency fluctuations from these data by calculating 3-point moving averages.

### One-box Os mass balance modelling

To explain the observed seawater (^187^Os/^188^Os)_*i*_ record, we calculated the Os mass balance and the Os isotope mass balance in the ocean and used them to constrain various scenarios. For simplicity, we assumed that the Os isotopic composition of the global ocean is homogenous, because the residence time of Os in the ocean is sufficiently longer than the period of global ocean circulation^12^. We then estimated the total amount of Os and its ^187^Os^/188^Os value in the ocean from the balance among the three Os fluxes into the ocean: continental input via rivers, mantle-derived input via volcanism and seafloor hydrothermal activity, and extra-terrestrial input from cosmic dust and meteorites (Supplementary Fig. S1). We employed a simple box model for the marine Os system based on these fluxes and the assumption that seawater Os is removed from the ocean into sediment. In this model, the mass balance and isotopic mass balance of Os can be expressed as follows^62^:

2 $$\begin{array}{*{20}c} {\frac{{dM_{{{\text{SW}}}} }}{dt} = F_{{{\text{riv}}}} + F_{{{\text{HT}}}} + F_{{{\text{cos}}}} - F_{{{\text{sed}}}} } \\ \end{array}$$

3 $$\begin{array}{*{20}c} {\frac{{{{dR}}_{{{\text{SW}}}} {{M}}_{{{\text{SW}}}} }}{{{{d}}t}} = {{F}}_{{{\text{riv}}}} {{R}}_{{{\text{riv}}}} + {{F}}_{{{\text{HT}}}} {{R}}_{{{\text{HT}}}} + {{F}}_{{{\text{cos}}}} {{R}}_{{{\text{cos}}}} - {{F}}_{{{\text{sed}}}} {{R}}_{{{\text{sed}}}} } \\ \end{array}$$

where *F* and *R* are the global fluxes (kg/yr) and globally averaged ^187^Os/^188^Os values for each flux, respectively, and *M*_SW_ represents the mass of Os in the ocean. The subscripts SW, riv, HT, cos, and sed indicate seawater, riverine, hydrothermal, and cosmic Os inputs, and sedimentary Os output, respectively. *R*_sed_ is assumed to be equivalent to *R*_SW_; that is, marine sediment is assumed to record the Os isotopic signature of the contemporary seawater without isotopic fractionation. By assuming that the output of Os in seawater to sediment is proportional to the total mass of Os in the ocean, *F*_sed_ can be related to *M*_SW_ by the proportionality factor *k*:

4 $$\begin{array}{*{20}c} {{{F}}_{{{\text{sed}}}} = {{kM}}_{{{\text{SW}}}} } \\ \end{array}$$

From Eqs. (2–4), we can derive following ordinary differential equations for the mass balance and isotopic mass balance of Os in seawater:

5 $$\begin{array}{*{20}c} {\frac{{{{dM}}_{{{\text{SW}}}} }}{{{{dt}}}} = {{F}}_{{{\text{riv}}}} + {{F}}_{{{\text{HT}}}} + {{F}}_{{{\text{cos}}}} - {{kM}}_{{{\text{SW}}}} } \\ \end{array}$$

6 $$\begin{array}{*{20}c} {\frac{{{{dR}}_{{{\text{SW}}}} }}{{{{dt}}}} = \frac{{{{F}}_{{{\text{riv}}}} \left( {{{R}}_{{{\text{riv}}}} - {{R}}_{{{\text{SW}}}} } \right) + {{F}}_{{{\text{HT}}}} \left( {{{R}}_{{{\text{HT}}}} - {{R}}_{{{\text{SW}}}} } \right) + {{F}}_{{{\text{cos}}}} \left( {{{R}}_{{{\text{cos}}}} - {{R}}_{{{\text{SW}}}} } \right)}}{{{{M}}_{{{\text{SW}}}} }}} \\ \end{array}$$

The parameter values used for these calculations are presented in Supplementary Table S1. Because there is no effective constraint on the steady state of the marine Os isotope system, we assume that the steady-state value of *M*_SW_ (*M*_SW_std_) is the modern value (1.37 × 10^7^ kg^12^). We tentatively set *R*_SW_ to the steady-state value (*R*_SW_std_) of ^187^Os/^188^Os = 1.006, which is the average observed seawater (^187^Os/^188^Os)_*i*_ value during 0–300 ka, instead of to the modern seawater value^14–16^ (^187^Os/^188^Os ~ 1.06). We used this average because recent research has suggested that the isotope system, including Os, might at present be in an imbalanced state^25^. Furthermore, because the estimated residence time of Os in the ocean is 25–45 kyr, it is possible that the marine Os isotope system has not completely recovered to a steady state since the last glacial maximum (LGM) at 14 ka.

The proportionality factor *k* was obtained by assuming that the marine Os isotope system is at steady state. First, we employed the modern Os fluxes of hydrothermal and cosmic dust as standard values for those fluxes (*F*_HT_std_, and *F*_cos_std_, respectively). We then used the following equation to estimate *F*_riv_ at steady state (*F*_riv_std_):

7 $$\begin{array}{*{20}c} {{{F}}_{{{\text{riv\_std}}}} = \frac{{\left( {{{R}}_{{{\text{SW\_std}}}} - {{R}}_{{{\text{HT}}}} } \right){{F}}_{{{\text{HT\_std}}}} + \left( {{{R}}_{{{\text{SW\_std}}}} - {{R}}_{{{\text{cos}}}} } \right){{F}}_{{{\text{cos\_std}}}} }}{{{{R}}_{{{\text{riv}}}} - {{R}}_{{{\text{SW\_std}}}} }}} \\ \end{array}$$

By using 1.006 as *R*_sw_std_ instead of the modern seawater ^187^Os/^188^Os value, *F*_riv_std_ was calculated to be 281 kg/yr, which is slightly lower than the estimated modern riverine Os flux based on observational data^63^ (*F*_riv_modern_ = 301 kg/yr, under the assumption that 15% of riverine Os is trapped in estuaries^64^). The difference between the riverine Os flux calculated by assuming a steady state (*F*_riv_std_) and the observed modern riverine flux (*F*_riv_modern_) can be attributed to the deviation of the modern marine Os isotope system from a steady state^25^. Then, we obtained *k* by the following equation using the *F*_riv_std_ value calculated above:

8 $$\begin{array}{*{20}c} {{{k}} = \frac{{{{F}}_{{{\text{riv\_std}}}} + {{F}}_{{{\text{HT\_std}}}} + {{F}}_{{{\text{cos\_std}}}} }}{{{{M}}_{{{\text{SW\_std}}}} }}} \\ \end{array}$$

As a result, *k* was calculated to be 0.028 kyr^−1^, and the residence time of Os in the ocean (*τ* = 1/*k*) was calculated to be 35 kyr, which is within the range of previous estimates (25–45 kyr)^12^. Therefore, we employed *τ* = 35 kyr as the default residence time value. However, because some research has suggested that the Os removal rate to sediment is variable owing to changes in the organic matter flux^65^, we also conducted the simulation using different residence times (*τ* = 25 kyr and 45 kyr) and, thus, different *k* values (*k* = 0.04 and 0.02, respectively).

To evaluate the cause of the observed *R*_SW_ fluctuation (assumed to be equal to *R*_sed_; Fig. 2b), we conducted simulations using Eqs. (5) and (6) and the calculated value of *k* to calculate *M*_SW_ and *R*_SW_ at 0.1 kyr timesteps, and tested possible scenarios by changing the *R*_riv_, *F*_riv_, and *F*_HT_ (see below) forcings. Because the Os flux of cosmic dust is thought to have been constant during the Quaternary^66,67^, we used the modern *F*_cos_ value in all simulations.

### Effect of riverine Os isotopic fluctuation

Because the global distribution of exposed lithology is variable, the Os isotopic composition of each drainage basin might be different. Moreover, the globally averaged ^187^Os/^188^Os value of river water was likely not constant throughout the Quaternary, because the exposed lithology probably differed between glacial and interglacial periods. During glacial intervals, the continental shields, which have large amounts of radiogenic Os (^187^Os/^188^Os =  ~ 2; ref.^68^), were covered by ice sheets in high-latitude areas, and continental shelf sediments, which have a relatively unradiogenic Os isotopic composition^69^ (^187^Os/^188^Os =  ~ 0.8), were exposed by falling sea levels. Both these phenomena likely caused changes in the globally averaged ^187^Os/^188^Os value of the riverine Os flux. Although it is difficult to verify this scenario because of the many factors that affect riverine ^187^Os/^188^Os values (e.g. global distribution of exposed lithology, precipitation patterns, and runoff rates), we roughly estimated the globally averaged riverine Os isotopic composition (*R*_riv_) in the present day and in the LGM from the global distribution of lithology^5^ with the following equation:

9 $$\begin{array}{*{20}c} {{{R}}_{{{\text{riv}}}} = \frac{{\mathop \sum \nolimits_{i} {{C}}_{i} {{A}}_{i} {{R}}_{i} }}{{\mathop \sum \nolimits_{i} {{C}}_{i} {{A}}_{i} }}} \\ \end{array}$$

where *A*, *C,* and *R* represent the exposed area, Os concentration, and ^187^Os/^188^Os value of each lithology, respectively, and the subscript *i* indicates the lithology: carbonates + shale, sandstones, extrusive igneous rocks (divided into basaltic + andesitic volcanic rocks and acidic volcanic rocks), shield rocks, or fold belts (complex lithology). The values used for this estimation and their sources are summarised in Supplementary Table S2. For carbonates + shale, ^187^Os/^188^Os = 1.78 (ref.^70^) was employed, because this value takes account of organic matter-containing sediments. The Os concentration in carbonate + shale was derived from the average Os concentrations of carbonate (8 ppt; ref.^69^) and shale (31 ppt; ref.^71^) weighted by the area fractions of carbonate and shale^72^. The area ratio of each lithology was estimated by using a 2° latitude by 2° longitude map of global lithology^5^**.**

According to the model result, the present value of ^187^Os/^188^Os in river waters is 1.43, and the value at the LGM was 1.41. Thus, the calculated difference in riverine ^187^Os/^188^Os values between glacial and interglacial intervals is < 1.6%. To verify the effect of changes of riverine ^187^Os/^188^Os values on the seawater ^187^Os/^188^Os value, we set the total Os input fluxes to the ocean to constant values (using *F*_riv_std_, *F*_HT_std_, and *F*_cos_std_; Supplementary Fig. S7b) and then allowed *R*_riv_ to fluctuate between 1.41 and 1.43, in synchrony with the LR04 δ^18^O record^22^, which represents ice sheet volume (Supplementary Fig. S7b).

The calculated seawater ^187^Os/^188^Os variations did not match those of the measured seawater (^187^Os/^188^Os)_*i*_ profile, and the amplitude of the variation was much smaller, even when we changed the residence time of Os to 25 kyr and 45 kyr (Supplementary Fig. S7a). Hence, we inferred that fluctuations of riverine ^187^Os/^188^Os were not a main driver of the glacial–interglacial marine ^187^Os/^188^Os fluctuations. This result suggests, therefore, that the observed seawater (^187^Os/^188^Os)_*i*_ profile mainly reflects the relative intensity of riverine and hydrothermal fluxes, even if the riverine ^187^Os/^188^Os value fluctuated because of changes in the exposed lithology on land. For simplicity, in the following discussion, we focus on riverine and hydrothermal Os fluxes under the assumption that the ^187^Os/^188^Os value of each influx is constant.

### Effect of pCO_2_ variations on the chemical weathering rate

It has been argued that the chemical weathering rate would have changed in association with glacial–interglacial cycles^17,18^. Under warm, humid interglacial conditions, the chemical weathering rate of silicate rocks would be enhanced, and under cold, dry glacial conditions, the rate would be suppressed. Assuming that the Os isotopic composition of each flux (*R*_riv_, *R*_HT_, and *R*_cos_) did not change and that the Os fluxes of hydrothermal and cosmic dust have been constant (*F*_HT_ = *F*_HT_std_; *F*_cos_ = *F*_cos_std_), we tested the scenario that the chemical weathering rate fluctuated in association with glacial–interglacial cycles. We estimated the silicate weathering rate to be a function of atmospheric *p*CO_2_ by using the LOSCAR carbon cycle model^73^ and assuming that *F*_riv_ directly reflects the silicate weathering rate. Thus, we used the following equation to estimate *F*_riv_ at each timestep.

10 $$\begin{array}{*{20}c} {{{F}}_{{{{riv}}}} = \left( {\frac{{{{p}}{{CO}}_{{{2}}} }}{{{{p}}{{CO}}_{{{{2}}_{{{{modern}}}} }} }}} \right)^{{{{n}}_{{{{Si}}}} }} \times {{F}}_{{{{riv}}_{{{{modern}}}} }} } \\ \end{array}$$

The *p*CO_2_ data used in the simulation were obtained from the Antarctic Vostok ice core record^74^ (Supplementary Fig. S8d). *F*_riv_ modern_ was set to the observed modern value (301 kg/yr). We used 0.2 (ref.^75^) as the default value of the feedback factor *n*_Si_, and we also changed *n*_*Si*_ within a reasonable range (from 0.2 to 0.6) to check the sensitivity of the result to its value. To calculate seawater ^187^Os/^188^Os (*R*_SW_), the *F*_riv_ value estimated with Eq. (10) was input into the Os mass balance model as a forcing for the past 400 kyr (Supplementary Fig. S8c). Before and after this forcing interval, *F*_riv_ was set to *F*_riv_std_ (281 kg/yr).

As a result, the fluctuations of the calculated *R*_SW_ profile when *n*_Si_ was set to 0.2 were smaller than those of the measured profile (Supplementary Fig. S8a, solid red line). Even when larger values of *n*_Si_ were used (*n*_Si_ = 0.4 and 0.6; Supplementary Fig. S8a, solid blue and orange lines), the fluctuations of the calculated profiles lagged those of the observed Os isotopic record, although the fluctuation amplitudes became larger. In addition, changing the Os residence time (to *τ* = 25 kyr or 45 kyr) did not affect the calculation result (dotted and dashed lines in Supplementary Fig. S7a). Therefore, we inferred that the *p*CO_2_ variation of ~ 100 ppm was too small to generate a significant change in the chemical weathering rate on a global scale. Even with a feedback factor *n*_Si_ larger than the default value (0.2), the *p*CO_2_ fluctuation could not be a main driver of seawater ^187^Os/^188^Os variation.

### Effect of including Os flux pulses in the model

As a final scenario to reproduce the measured seawater Os isotope fluctuation, we added two mechanisms that could cause seawater ^187^Os/^188^Os to be (1) more radiogenic during interglacials and (2) more unradiogenic during glacials to the scenario where the silicate chemical weathering rate is synchronous with *p*CO_2_ fluctuation. Therefore, we input pulses of large amounts of radiogenic and unradiogenic Os to the model as forcings.

We hypothesised that radiogenic Os pulses might be generated by intense chemical weathering of fresh, finely ground clastic sediments (glacial till) during deglacial periods, as argued previously^24,25^, and that unradiogenic Os pulses might be generated by enhanced hydrothermalism triggered by falling sea levels during glacial intervals^7,26–29^.

We tested the scenario where (1) the riverine Os pulse input during each deglaciation was a forcing in addition to the background riverine Os flux that was synchronised with *p*CO_2_ fluctuations (see previous section) that caused the seawater Os isotopic composition to become more radiogenic, and (2) the hydrothermal pulse input, which coincided with minimal RSL values, was in addition to the constant hydrothermal Os flux. We searched for the best fit, the model with the minimum root mean squared error (RMSE) between observed and modelled seawater ^187^Os/^188^Os, by changing the magnitude of each pulse.

The riverine Os pulses were timed to coincide with each glacial termination (11, 61, 132, and 243 ka^31^), and the hydrothermal Os pulses were timed to coincide with minimal RSLs (20, 64, 88, 110, 154, 180, 205, 228, 273, and 300 ka^31^). The criterion for a decrease in RSL sufficient to cause a hydrothermal Os pulse was a RSL fall of more than 20 m compared to the previous RSL. Because we could not constrain Os input pulse magnitudes, we changed the magnitudes of each riverine and hydrothermal Os pulse from 1.0 × 10^5^ to 20 × 10^5^ kg and from 1.0 × 10^5^ to 10 × 10^5^ kg, respectively, in steps of 1 × 10^5^ kg. Because the chemical weathering rate should be highest immediately after glacial till becomes exposed^8,25^, the riverine pulse profile was assumed to follow the exponential law, and the duration of each pulse was set to 20 kyr. The duration of each hydrothermal pulse was also set to 20 kyr, and the input was set to have a Gaussian profile, to match the shape of the Fe flux excursion of hydrothermal sediments during the LGM^27,28^. We used a local search algorithm with Python for model optimisation. Because it is difficult to obtain a global optimum solution by using a local search algorithm, we repeated the model calculations 1,000 times and adopted the set of parameter values that produced the minimum RMSE as the best fit. Each calculation was terminated when the RMSE value had remained constant for 200 iterations.

As sensitivity analyses, we also carried out calculations including (1) only riverine Os pulses or (2) only hydrothermal Os pulses. However, the modelled results did not match with the observed seawater (^187^Os/^188^Os)_*i*_ record (Supplementary Fig. S9; RMSE > 0.00712). The calculated results were not affected even by the use of Os residence times in the ocean of *τ* = 25 kyr and 45 kyr.

In the best-fit scenario, RMSE was minimal (0.00676) when the magnitudes of the deglacial riverine Os pulses were 7 × 10^5^ kg (11 ka), 6 × 10^5^ kg (61 ka), 3 × 10^5^ kg (132 ka), and 1 × 10^5^ kg (243 ka), and the magnitudes of the hydrothermal-derived Os pulses injected into the ocean were 2 × 10^5^ kg (20 ka), 7 × 10^5^ kg (64 ka), 5 × 10^5^ kg (88 ka), 4 × 10^5^ kg (110 ka), 2 × 10^5^ kg (154 ka), 3 × 10^5^ kg (180 ka) , 6 × 10^5^ kg (205 ka), 3 × 10^5^ kg (228 ka), 4 × 10^5^ kg (273 ka), and 1 × 10^6^ kg (300 ka) (Fig. 4c, d, solid blue lines). Convergence was typically obtained after 700 iterations (Supplementary Fig. S10). The calculated *R*_SW_ profile was consistent with the observed seawater (^187^Os/^188^Os)_*i*_ pattern obtained from the ODP Site 834A core (Fig. 4b, solid blue line). Therefore, we concluded that this scenario was the most plausible explanation for the marine Os isotope record.

Because the residence time of Os may be variable^65^, we also conducted these calculations using different *τ* values. When the residence time was shorter than the default value (*τ* = 25 kyr), the RMSE was minimal (0.00764) when the magnitudes of the deglacial riverine Os pulses were 4 × 10^5^ kg (11 ka), 2 × 10^5^ kg (61 ka), 1 × 10^5^ kg (132 ka), and 1 × 10^5^ kg (243 ka), and the magnitudes of the hydrothermal-derived Os pulses were 2 × 10^5^ kg (20 ka), 5 × 10^5^ kg (64 ka), 4 × 10^5^ kg (88 ka), 4 × 10^5^ kg (110 ka), 2 × 10^5^ kg (154 ka), 3 × 10^5^ kg (180 ka), 5 × 10^5^ kg (205 ka), 4 × 10^5^ kg (228 ka), 4 × 10^5^ kg (273 ka), and 8 × 10^5^ kg (300 ka) (Fig. 4c, d, dotted blue lines). On the other hand, when the residence time was longer than the default value (*τ* = 45 kyr), the RMSE was minimal (0.00668) when the magnitudes of the deglacial riverine Os pulses were 1 × 10^6^ kg (11 ka), 8 × 10^5^ kg (61 ka), 5 × 10^5^ kg (132 ka), and 1 × 10^5^ kg (243 ka), and the magnitudes of the hydrothermal-derived Os pulses were 2 × 10^5^ kg (20 ka), 8 × 10^5^ kg (64 ka), 5 × 10^5^ kg (88 ka), 5 × 10^5^ kg (110 ka), 2 × 10^5^ kg (154 ka), 2 × 10^5^ kg (180 ka), 7 × 10^5^ kg (205 ka), 3 × 10^5^ kg (228 ka), 4 × 10^5^ kg (273 ka), and 8 × 10^5^ kg (300 ka) (Fig. 4c, d, dashed blue lines). Both scenarios (*τ* = 25 kyr and 45 kyr) showed different magnitudes of Os pulses to some extent, compared with the result obtained by using the default residence time value (*τ* = 35 kyr). However, all of the results support the conclusion that the coupling of rapid inputs of Os via glacial-till weathering and enhanced hydrothermalism can explain the (^187^Os/^188^Os)_*i*_ record from Site 834A.

## Supplementary Information

Supplementary Information 1.

Supplementary Information 2.

Supplementary Information 3.

Supplementary Information 4.

Supplementary Information 5.

## Data Availability

Source data for Figs. 2, 3, and 4 (and the Supplementary Figures containing data graphs) are provided with the paper.

## Code availability

The Python code for calculating the osmium isotopic mass-balance simulation is available from the corresponding author upon reasonable request.
