# Supplementary material for: Rapid coupling between solid earth and ice volume during the Quaternary
Source: Sci Rep. 2021 Mar 11;11:5695. doi: 10.1038/s41598-021-84448-7 (PMC7970951; doi:10.1038/s41598-021-84448-7)
Supplement: Supplementary file 5 — Supplementary Information 5. [file 41598_2021_84448_MOESM5_ESM.pdf]

Supplementary Information for

Rapid coupling between solid earth and ice volume during the Quaternary

Yusuke Kuwahara, Kazutaka Yasukawa, Koichiro Fujinaga, Tatsuo Nozaki, Junichiro Ohta, Honami Sato, Jun-Ichi Kimura, Kentaro Nakamura, Yusuke Yokoyama, Yasuhiro Kato

Correspondence to: ykato@sys.t.u-tokyo.ac.jp

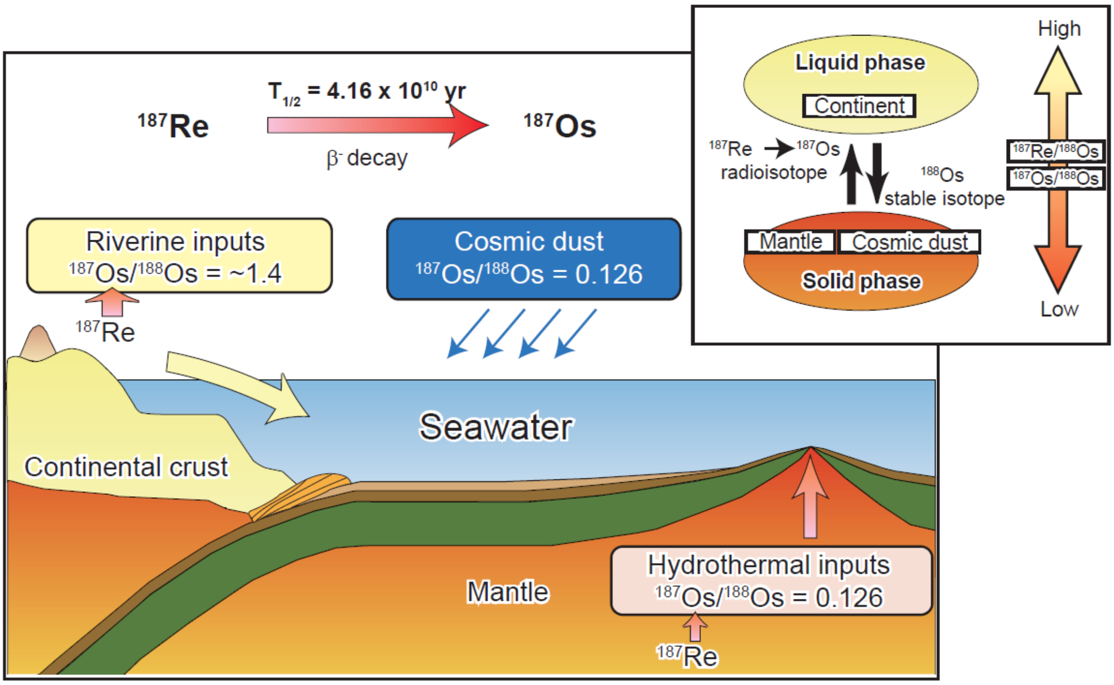

Supplementary Figure S1. Schematic illustration of the marine Os isotope system. Seawater ^{187}Os/^{188}Os is controlled by the balance of continental riverine, hydrothermal, and cosmic dust inputs. Because Re is less compatible than Os in partial melts, ^{187}Re, the parent nuclide of ^{187}Os, is enriched in the liquid phase (which becomes continental crust), whereas unradiogenic ^{188}Os is relatively more concentrated in the solid phase (i.e. in mantle-like materials). As a result, ^{187}Os/^{188}Os values of continental-derived materials are higher than those of mantle-like material (hydrothermal fluid and cosmic dust).

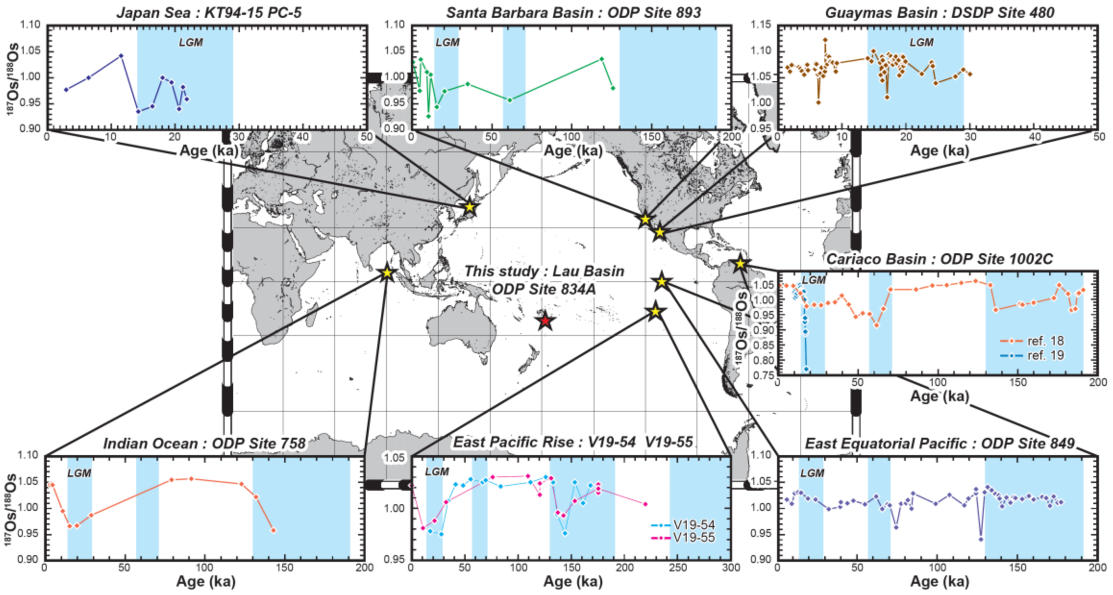

Supplementary Figure S2. Compilation of previously published data[17–19,76–78] used for the reconstruction of the Quaternary seawater $^{187}Os/^{188}Os$ record. Each record shows initial $^{187}Os/^{188}Os$ values. Vertical blue bars indicate glacial periods (marine isotope stages 2, 4, 6, and 8)[22]. Although almost all of these studies show a negative excursion of $^{187}Os/^{188}Os$ during the last glacial maximum (LGM), the profiles are not otherwise consistent among the studies. Data sources: V19-54, V19-55 (ref. 17); ODP Site 1002C (refs. 18, 19); DSDP Site 480 (ref. 19); ODP Site 849 (ref. 19); ODP Site 893 (ref. 76); KT94-15 PC-5 (ref. 77); ODP Site 758 (ref. 78). The world map is generated by the Generic Mapping Tools (GMT Version 6.0.0, https://github.com/GenericMappingTools/gmt/releases/tag/6.0.0).

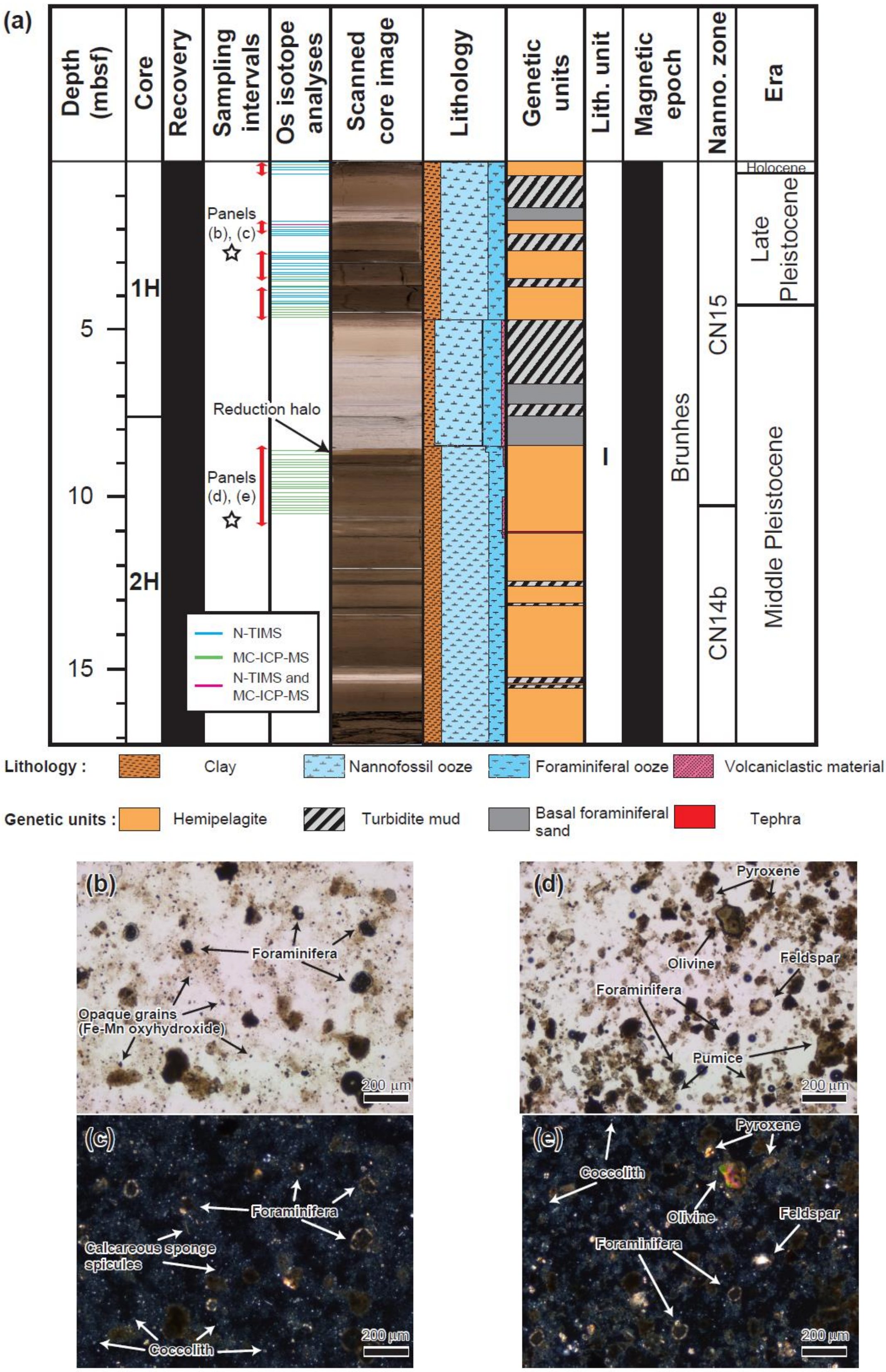

(a)
Depth (mbsf)
Core
Recovery
Sampling intervals
Os isotope analyses
Scanned core image
Lithology
Genetic units
Lith. unit
Magnetic epoch
Nanno. zone
Era
5
10
15
1H
2H
Panels (b), (c)
Panels (d), (e)
Reduction halo
N-TIMS
MC-ICP-MS
N-TIMS and MC-ICP-MS
I
Brunhes
CN15
CN14b
Holocene
Late Pleistocene
Middle Pleistocene
Lithology :
Clay
Nannofossil ooze
Foraminiferal ooze
Volcaniclastic material
Genetic units :
Hemipelagite
Turbidite mud
Basal foraminiferal sand
Tephra
(b)
Foraminifera
Opaque grains (Fe-Mn oxyhydroxide)
200 µm
(c)
Foraminifera
Calcareous sponge spicules
Coccolith
200 µm
(d)
Pyroxene
Olivine
Feldspar
Foraminifera
Pumice
200 µm
(e)
Pyroxene
Coccolith
Olivine
Feldspar
Foraminifera
200 µm

Supplementary Figure S3. Core lithology and smear slide microphotographs. (a) Lithostratigraphy, genetic stratigraphy, and core photograph of the ODP Site 834A cores 1H and 2H (modified from refs. 46–48). Magnetostratigraphic epoch, biostratigraphic zone, and depositional era[47,48] are shown alongside. Double-headed red arrows in the "sampling intervals" column indicates the sampling ranges from which we picked samples for foraminiferal $\delta^{18}O$ analyses (n = 125). We avoided sampling the turbiditic zones. For the bulk chemical and Re-Os isotope analyses, we used sediments from shallower than 10.4 mbsf in the core (n = 115) to avoid contamination with volcaniclastic components, which increase downward in the core. Bars in the Os isotope analyses column indicate sampling points for the analyses. Light blue and green bars indicate sampling points for analyses by N-TIMS and MC-ICP-MS, respectively. The magenta bar indicates the sampling point for duplicate analysis, by both N-TIMS and MC-ICP-MS. The light orange facies in the core photograph (black arrow) is the reduction halo. (b–e) Microphotographs of smear slide samples of sediments from ODP Site 834A. (b, c) Metalliferous carbonate (Core 1H, Section 2, 118–120 cm; 2.69 mbsf) under (b) plane- and (c) cross-polarised light. (d, e) Sediment containing volcaniclastic materials from ODP Site 834A (Core 2H, Section 3, 9–11 cm; 10.7 mbsf) under (d) plane- and (e) cross-polarised light.

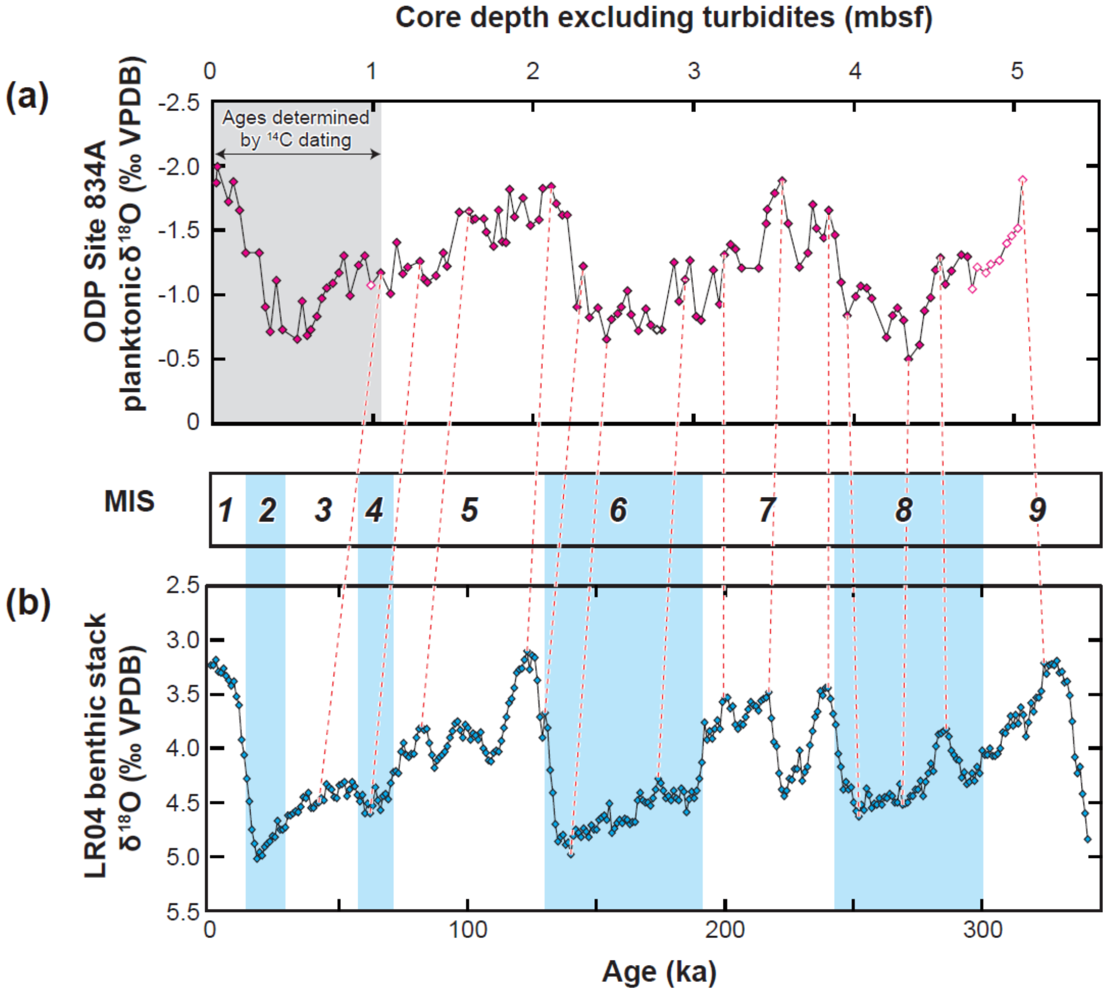

Supplementary Figure S4. Correlation between (a) δ^{18}O data from ODP Site 834A and (b) the reference δ^{18}O profile (LR04 benthic stack[22]). Grey shading indicates the age range of samples dated by radiocarbon analysis. The inflection points of the curves were determined in this study. Turbidite intervals have been omitted. Vertical blue bars indicate glacial periods, and the numerals indicate MISs. The solid magenta diamonds in (a) indicate samples analysed for both δ^{18}O and bulk chemistry. The open magenta diamonds in (a) indicate samples analysed only for δ^{18}O. The open black square at 2.77 mbsf in (a) indicates the sample for which we could not calculate the standard deviation of δ^{18}O.

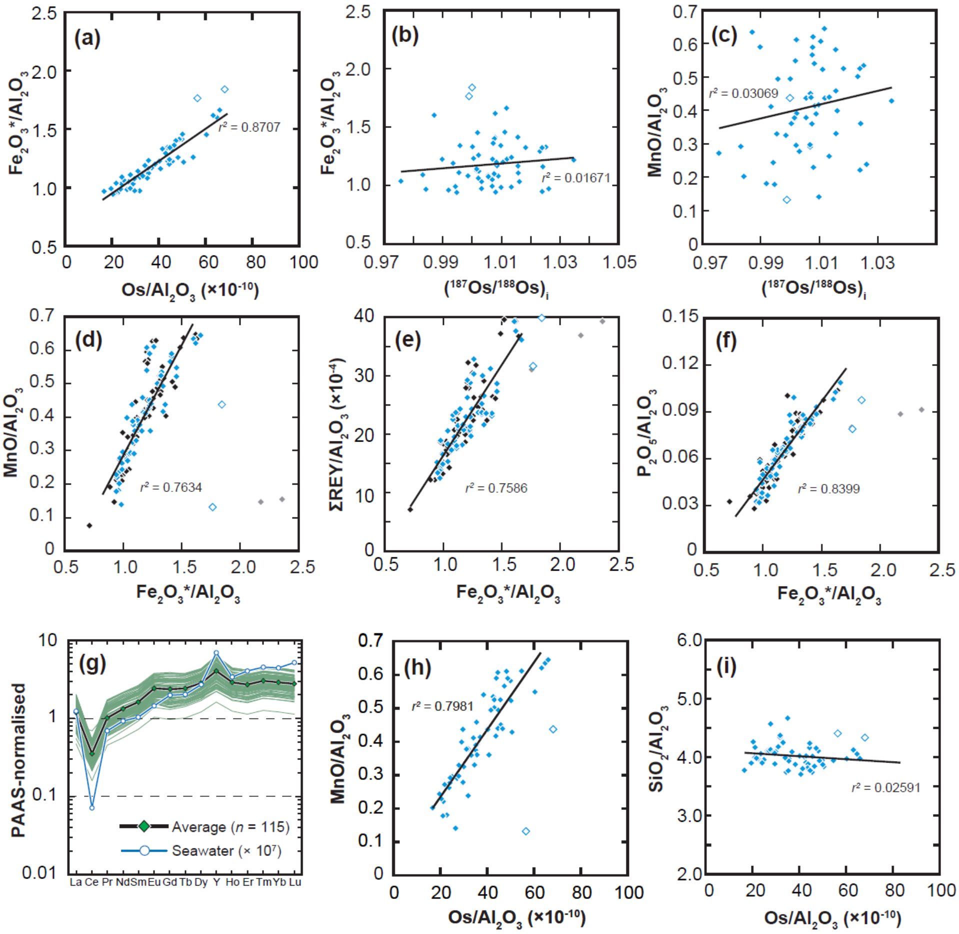

Supplementary Figure S5. Geochemical data of ODP Site 834A sediments. Panels (a), (h), and (i) show Os versus Fe_2O_3* (total iron as Fe_2O_3), MnO, and SiO_2, respectively. Panels (b) and (c) show $(^{187}Os/^{188}Os)_i$ versus Fe_2O_3* and MnO, respectively. Panels (d), (e), and (f) show Fe_2O_3* versus MnO, total rare-earth elements + Y (ΣREY), and P_2O_5 contents, respectively. The contents of all elements are normalised by the Al_2O_3 content and were calculated on a carbonate-free basis (CFB). The solid blue diamonds in panels (a) to (f) and (h) to (i) indicate samples used for Re-Os isotope analyses, and black diamonds indicate samples not used for Re-Os analyses. Samples collected from the reduction halo (see Methods) are indicated by light blue open diamonds (Re-Os analysed) or grey diamonds. Linear regression lines and r^2 values were calculated by using all data except data from the reduction halo samples. Panel (g) shows patterns of REY calculated on a CFB and normalised by the post-Archean average Australian shale (PAAS[51]). The black line with green diamonds shows the average of all samples (n = 115). The blue line with open blue circles shows the REY pattern of seawater ($\times 10^7$)[79].

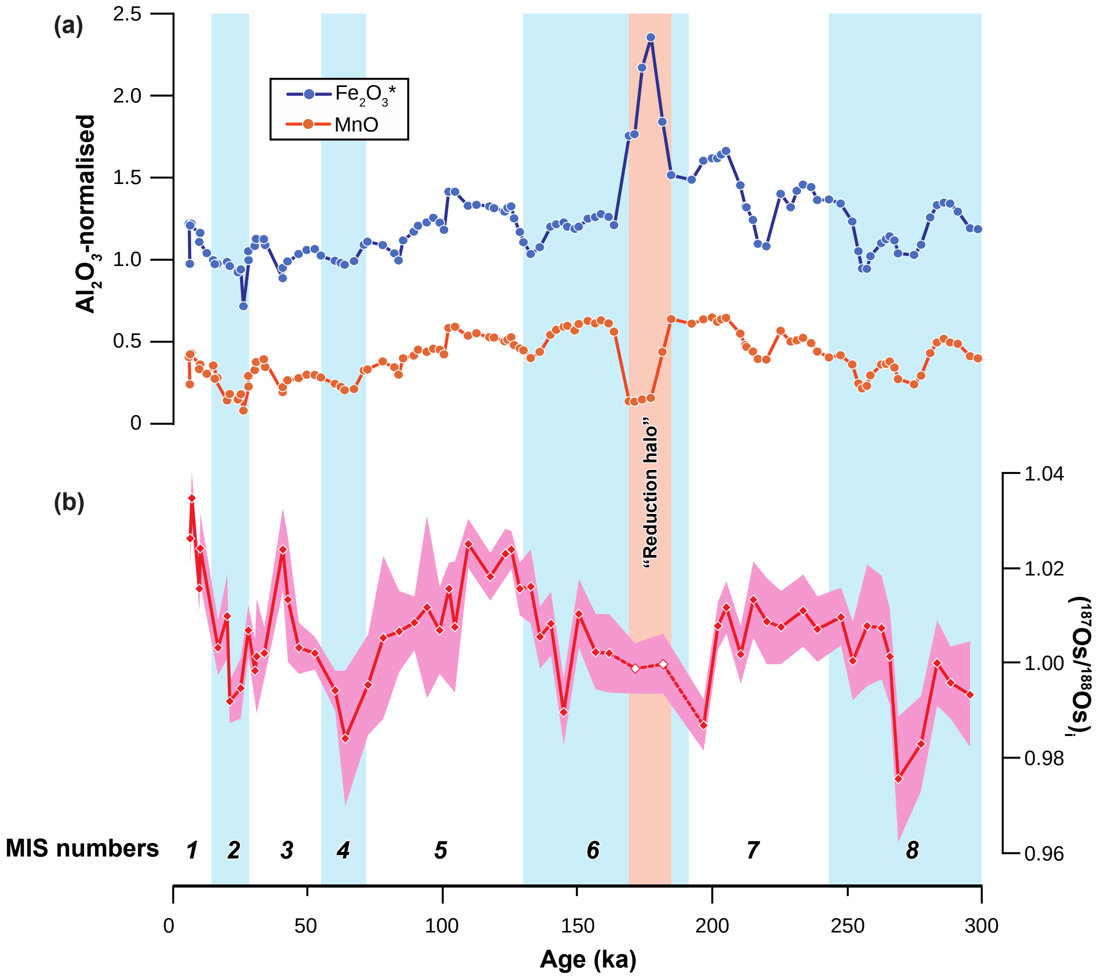

Supplementary Figure S6. Age profiles of (a) Fe_2O_3* (total iron as Fe_2O_3) and MnO contents, and (b) the $(^{187}Os/^{188}Os)_i$ record in ODP Site 834A sediments. In panel (a), the Fe_2O_3* and MnO contents, which were calculated on a carbonate-free basis (CFB), are normalised by the Al_2O_3 content. The red shaded area in panel (b) indicates the 2SD range of $(^{187}Os/^{188}Os)_i$. The vertical orange bar indicates the reduction halo[23], which is developed below the turbiditic layer. Vertical blue bars indicate glacial periods (MIS 2, 4, 6, 8).

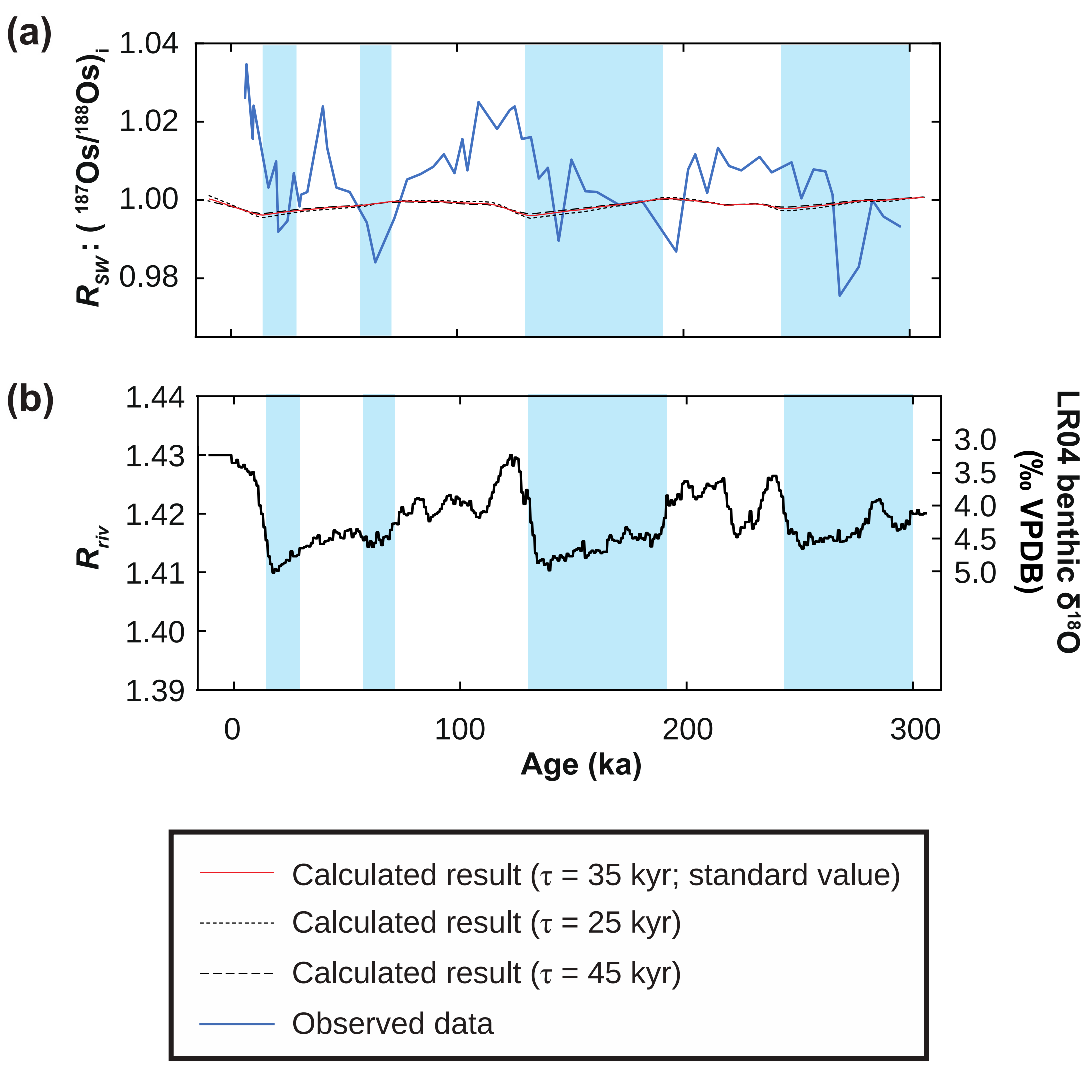

Supplementary Figure S7. Simulated seawater (^{187}Os/^{188}Os)$_i$ under the scenario that the riverine Os isotopic composition fluctuated in association with glacial–interglacial cycles. (a) Calculated Os isotopic ratio of seawater (R_{SW}), and (b) Os isotopic ratio of river water (R_{riv}) imposed on the model. Each Os flux (riverine, hydrothermal, and cosmic) was set to a constant value. R_{riv} was made to fluctuate between 1.43 and 1.41 in synchrony with the LR04 benthic stack[22], reflecting the growth and regression of ice sheets (b). In panel (a), the results of calculations using Os residence times in the ocean of τ = 25 kyr and 45 kyr are also shown. The fluctuations of calculated R_{SW} are small under this scenario, and they do not correspond to the variations in the observed data (a). Vertical blue bars indicate glacial periods.

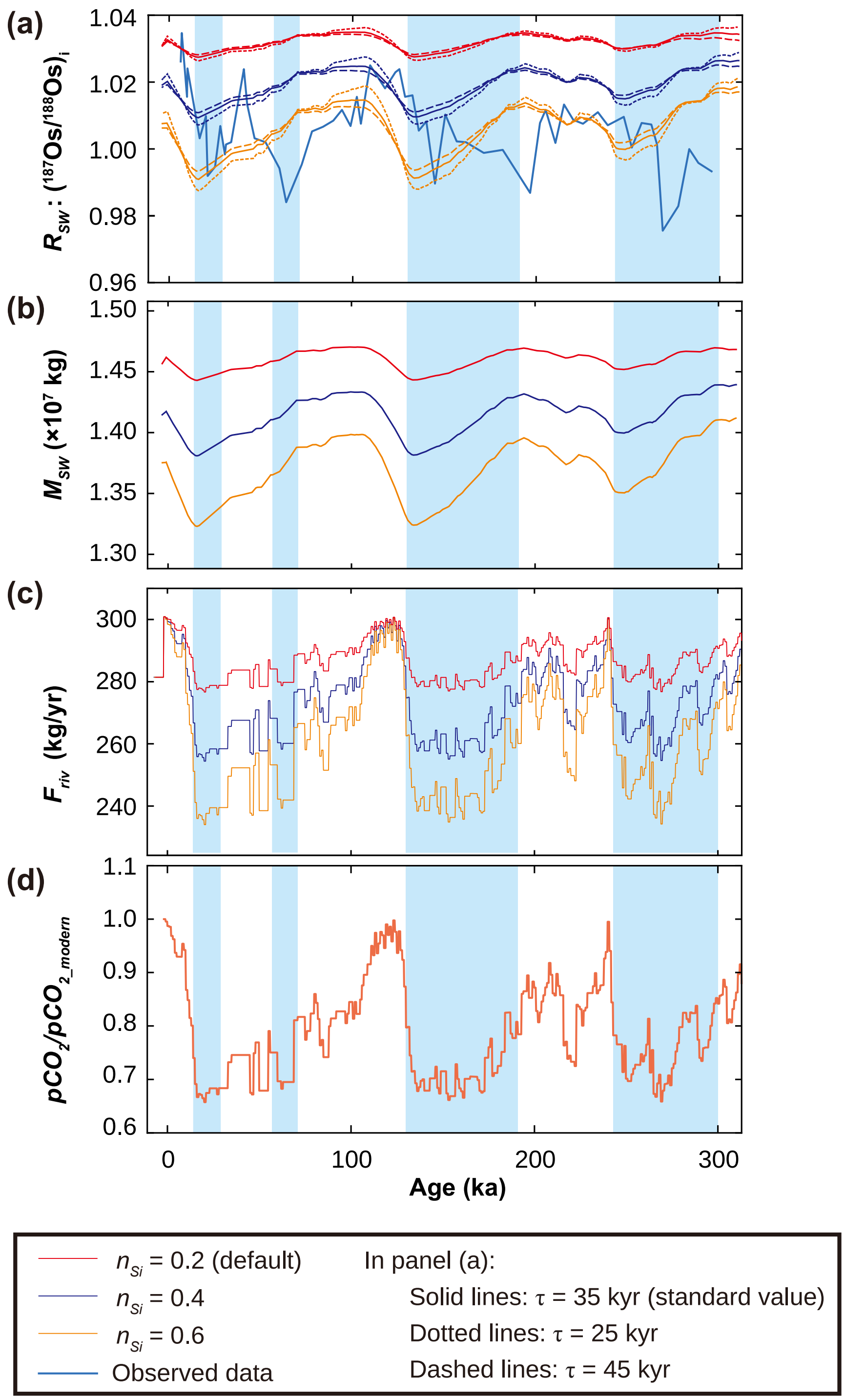

Supplementary Figure S8. Simulated seawater (^{187}Os/^{188}Os)$_i$ under the scenario that the riverine Os flux varied in association with glacial–interglacial cycles. (a) Calculated Os isotopic ratio of seawater (R_{SW}), (b) seawater Os inventory (M_{SW}), (c) riverine Os flux (F_{riv}) imposed on the model, and (d) pCO$_2$ data from the Vostok ice core[68]. The ^{187}Os/^{188}Os value of each Os flux (riverine, hydrothermal, and cosmic) was assumed

to be constant. F_{riv} fluctuated along with pCO_2 (d). The n_{Si} value (silicate weathering parameter, see Methods) was set at 0.2 (default, red lines), and then at 0.4 (blue lines) and 0.6 (orange lines). With n_{Si} = 0.2 (panel a, red lines), the calculated R_{SW} fluctuations did not show any relationship to the observed data. Even with n_{Si} = 0.4 or 0.6, fluctuations in the calculated profiles did not correspond to variations in the observed $^{187}Os/^{188}Os$ profile. In panel (a), solid lines, dotted lines, and dashed lines indicate the calculation results obtained using τ = 35 kyr (default), 25 kyr, and 45 kyr, respectively. Vertical blue bars indicate glacial periods[22].

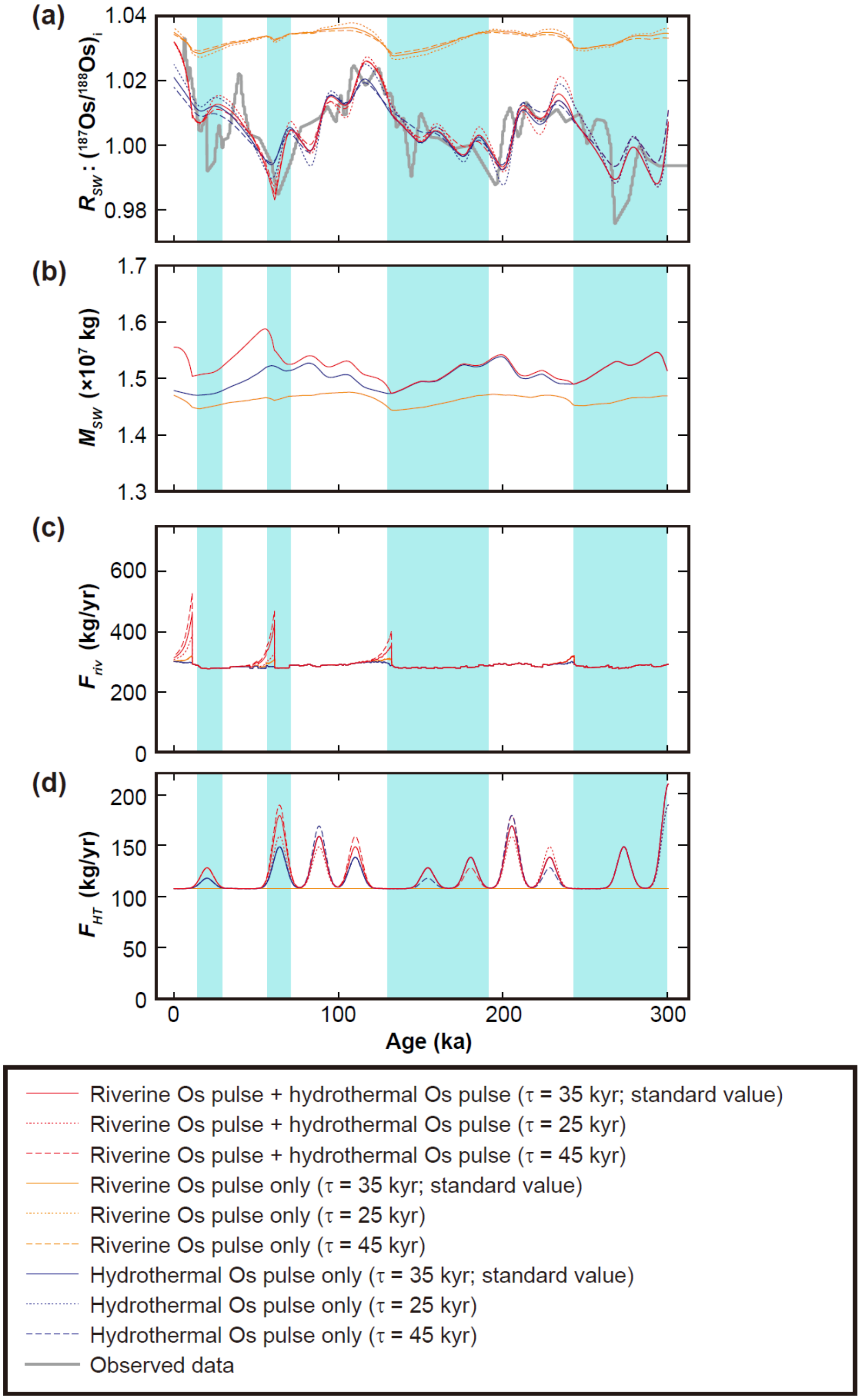

Supplementary Figure S9. Simulated seawater ($^{187}Os/^{188}Os)_i$ under the scenario that includes riverine and hydrothermal Os pulses. (a) Calculated Os isotopic ratio of seawater (R_{SW}), (b) seawater Os inventory (M_{SW}), (c) riverine Os flux (F_{riv}) imposed on the model, and (d) hydrothermal Os flux (F_{HT}) imposed on the model. The $^{187}Os/^{188}Os$

value of each Os flux (riverine, hydrothermal, and cosmic) was assumed to be constant. As sensitivity analyses, we also carried out calculations including only the riverine Os pulse (orange lines) or only the hydrothermal Os pulse (blue lines). Vertical blue bars indicate glacial periods[22]. In panels (a), (c), and (d), solid lines, dotted lines, and dashed lines indicate the calculation results obtained using τ = 35 kyr (default), 25 kyr, and 45 kyr, respectively.

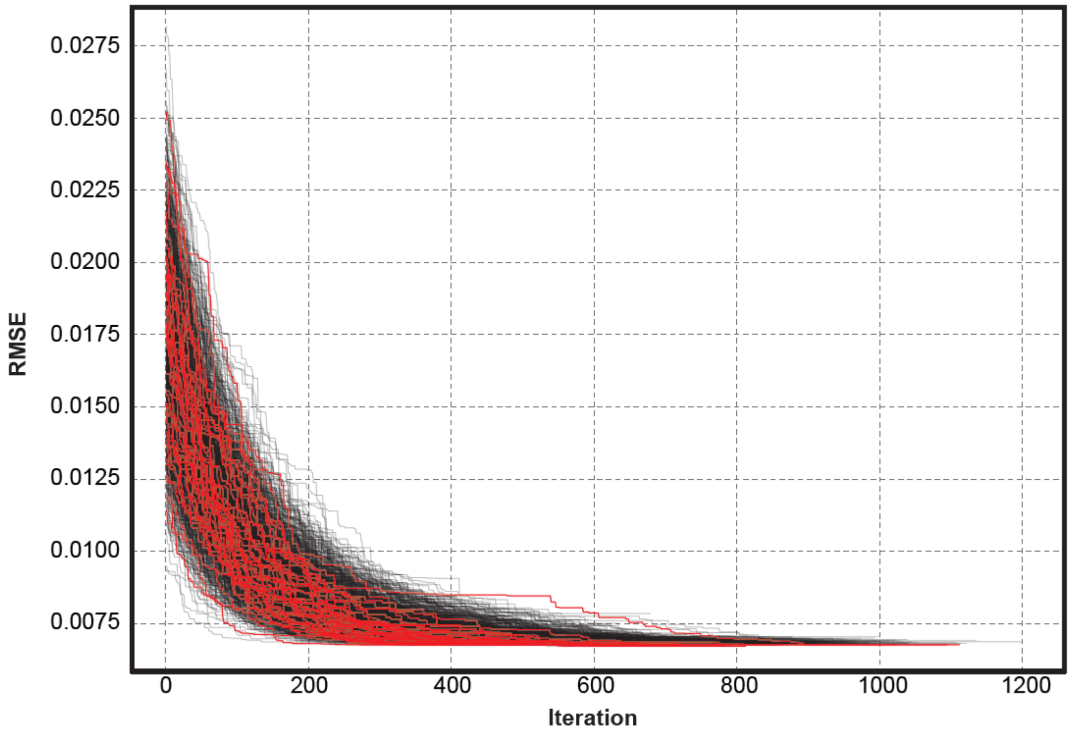

Supplementary Figure S10. Converging trajectories of RMSE values between observed data and results modelled by using a local search algorithm. Thin black lines show the converging behaviour of each local search algorithm calculation (*n* = 1,000). Red lines show the converging behaviour of the calculations that generated the minimum RMSE value (0.00676) among the 1,000 calculations.

Supplementary Table S1. Parameters used in the mass balance calculation.

	Symbol	Value	Unit	Remarks
Mass of Os in seawater at steady state	M_{SW_std}	1.37×10^{7}*	kg	Value of modern ocean
$^{187}Os/^{188}Os$				
Seawater at steady state	R_{SW_std}	1.006		Average value of observed $^{187}Os/^{188}Os$ record
Riverine water	R_{riv}	1.43*		In the calculation verifying the effect of Os isotopic fluctuation, we fluctuated R_{riv} between 1.43 and 1.41
Hydrothermal fluid	R_{HT}	0.126†		
Cosmic dust	R_{cos}	0.126†		
Os flux				
Modern riverine flux	F_{riv_modern}	301‡	kg/yr	Trapped fraction at estuaries (15%; ref. 64) was considered
Riverine flux (standard value)	F_{riv_std}	281	kg/yr	Calculated from eq. (6). Trapped fraction at estuaries (15%; ref. 64) was considered
Hydrothermal flux (standard value)	F_{HT_std}	108†	kg/yr	
Cosmic flux (standard value)	F_{cos_std}	17.6†	kg/yr	
Silicate minerals chemical weathering exponent	n_{Si}	0.2‖		In the sensitivity analyses, the value was changed to 0.4 and 0.6

*Peucker-Ehrenbrink & Ravizza (2000)[12]; †Levasseur et al. (1999)[32]; ‡Peucker-Ehrenbrink (2002)[63]; ‖ Uchikawa & Zeebe (2008)[75]

Supplementary Table S2. Area fractions and bulk rock Os isotope ratios of each lithology used in the calculation of the Os isotopic ratio of riverine water.

Lithology		Area percentage of total ice free area*		[Os] (ppt)	$^{187}Os/^{188}Os$	Remarks
		Modern	LGM			
Carbonates+shales		27.3	31.9	25 (modern), 24 (LGM)#	1.78†	Area fractions of carbonates+shales and sandstones are based on ref. 72
Sandstones		18.6	20.2	31 (16–100)‡	1 (0.875–1.564)‡	Using [Os] and $^{187}Os/^{188}Os$ value of loess
Extrusive igneous rocks	Basaltic+andesitic volcanic rocks	5.8§	5.5	3.9 (0.104–46)‖	0.39 (0.130–2.302)‖	Based on the average [Os] and $^{187}Os/^{188}Os$ values of arc volcanic rocks presented by refs. 81,82.
	Acidic volcanic rocks	1§	1	1.3 (0.048–7.781)‖	1.04 (0.155–3.704)‖	
Shields		20	15.7	21¶	2.4¶	Based on the [Os] and $^{187}Os/^{188}Os$ values of a sample from the Canadian Shield (CAN96-31)
Fold belts (complex lithology)		27.5	25.7	31 (16–100)‡	1‡	Using [Os] and $^{187}Os/^{188}Os$ value of loess

*Gibbs & Kump (1994)[5]; †Li et al. (2009)[70]; ‡Peucker-Ehrenbrink & Jahn (2000)[71]; §Dürr et al. (2005)[80]; ‖Alves et al. (1999)[81]; Alves et al. (2002)[82]; ¶ Huh et al. (2004)[68]

#The average Os concentrations of carbonate (8 ppt; ref. 69) and shale (31 ppt, ref. 71) weighted by the area fractions of carbonate and shale.

Additional References in Supplementary Information

76. Williams, G. A. & Turekian, K. The glacial–interglacial variation of seawater osmium isotopes as recorded in Santa Barbara Basin. *Earth Planet. Sci. Lett.* **228**, 379-389 (2004).
77. Dalai, T. K., Suzuki, K., Minagawa, M. & Nozaki, Y. Variations in seawater osmium isotope composition since the last glacial maximum: a case study from the Japan Sea. *Chem. Geol.* **220**, 303-314 (2005).
78. Burton, K. W., Gannoun, A. & Parkinson, I. J. Climate driven glacial–interglacial variations in the osmium isotope composition of seawater recorded by planktic foraminifera. *Earth Planet. Sci. Lett.* **295**, 58-68 (2010).
79. Alibo, D. S., & Nozaki, Y. Rare earth elements in seawater: particle association, shale-normalization, and Ce oxidation. *Geochim. Cosmochim. Acta*, **63**, 363-372 (1999).
80. Dürr, H. H., Meybeck, M., & Dürr, S. H. Lithologic composition of the Earth's continental surfaces derived from a new digital map emphasizing riverine material transfer. *Glob. Biogeochem. Cycles*, **19**, GB4S10 (2005).
81. Alves, S., Schiano, P. & Allègre, C. J. Rhenium–osmium isotopic investigation of Java subduction zone lavas. *Earth Planet. Sci. Lett.* **168**, 65-77 (1999).
82. Alves, S., Schiano, P., Capmas, F., & Allègre, C. J. Osmium isotope binary mixing arrays in arc volcanism. *Earth Planet. Sci. Lett.* **198**, 355-369 (2002).
